# Correcting for Antibody Waning in Cumulative Incidence Estimation From Sequential Serosurveys

**DOI:** 10.1093/aje/kwad226

**Published:** 2023-11-27

**Authors:** Sarah Kadelka, Judith A Bouman, Peter Ashcroft, Roland R Regoes

**Keywords:** cumulative incidence, infectious disease, SARS-CoV-2, serosurveys

## Abstract

Serosurveys are a widely used tool to estimate the cumulative incidence—the fraction of a population that has been infected by a given pathogen. These surveys rely on serological assays that measure the level of pathogen-specific antibodies. Because antibody levels are waning, the fraction of previously infected individuals that have seroreverted increases with time past infection. To avoid underestimating the true cumulative incidence, it is therefore essential to correct for waning antibody levels. We present an empirically supported approach for seroreversion correction in cumulative incidence estimation when sequential serosurveys are conducted in the context of a newly emerging infectious disease. The correction is based on the observed dynamics of antibody titers in seropositive cases and validated using several in silico test scenarios. Furthermore, through this approach we revise a previous cumulative incidence estimate relying on the assumption of an exponentially declining probability of seroreversion over time, of severe acute respiratory syndrome coronavirus 2, of 76% in Manaus, Brazil, by October 2020 to 47.6% (95% confidence region: 43.5–53.5). This estimate has implications, for example, for the proximity to herd immunity in Manaus in late 2020.

## Abbreviations

anti-N: anti–nucleocapsid protein
CR: confidence region
IgG: immunoglobulin G
IgM: immunoglobulin M
SARS-CoV-2: severe acute respiratory syndrome coronavirus 2
S/C ratio: signal-to-cutoff

##  

Understanding how much an emerging infectious disease has spread in a population or geographical region is critical for the estimation of epidemiologic parameters such as the case ascertainment rate or infection fatality rate, which consequently influence decision making on measures undertaken to contain the disease (1). One important tool for assessing the cumulative incidence of an infectious disease are serosurveys, in particular when a large fraction of infections pass symptom-free and hence remains undetected (2–4).

In serosurveys, depending on the purpose and context of the study, the presence of antibodies against a certain antigen is used as a marker for past infection, vaccination or immunity (5). Serosurveys have been conducted for a wide range of antigens, such as the measles and rubella viruses (5) and the Zika virus (6, 7). A large body of serosurveys have also been performed to estimate the seroprevalence of severe acute respiratory syndrome coronavirus 2 (SARS-CoV-2) in various regions of the world (8).

Antibody persistence ranges from lifelong for some viral infections, such as measles and yellow fever (9), to a relatively fast decay for other viral infections, such as West Nile (10), seasonal coronaviruses (11), and SARS-CoV-2 (12–14). Rapidly decaying antibody levels result in an increasing likelihood of seronegativity over time despite prior antigen exposure. This transition of antibody levels from above a positivity threshold (seropositive) to below the threshold (seronegative) is called seroreversion and should be corrected for when estimating cumulative incidence from serological surveys. This correction gains in importance as the time from first antigen exposure in the population to the serosurvey grows. The lack of such correction can result in significant underestimates of cumulative incidence. Prior to the current SARS-CoV-2 pandemic, one study considering measles-mumps-rubella vaccine coverage in Australia corrected for seroreversion by assuming fixed seroreversion rates for all 3 antibodies. These rates, in addition to the vaccine coverage, were fitted using serial serosurvey data (15). Another study estimated the temporal immunoglobulin M (IgM) antibody profile in West Nile virus–infected individuals to derive time-dependent estimates for the probability of IgM-seropositivity (16). These, in combination with the temporal profile of reported cases, were used to estimate the cumulative incidence of the West Nile virus infection in the North Texas region, during the 2012 epidemic, from serological survey data.

Many serological studies have been conducted in the current SARS-CoV-2 epidemic (8). However, despite the wide-spread acknowledgement of the importance of seroreversion correction (17–19), only few studies have actually done so (19–24). In one such study, Buss et al. (23) estimated a cumulative incidence of 76% (95% confidence interval: 66.6, 97.9) in Manaus, Brazil, by October 2020 compared with an uncorrected 25.8% (95% confidence interval: 20.9, 31.3) using data from monthly serosurveys between March and October 2020. While this cumulative incidence should have conferred herd immunity and curbed the epidemic (25–27), Manaus was hit by a very strong second wave in January 2021 (28, 29). Multiple explanations for these puzzling patterns have been proposed: methodological issues relating to cumulative incidence estimation, waning of immunity, and new viral variants that evade immunity from previous infection or have increased transmissibility compared with the initially circulating variant (28).

Here, we describe a cutoff-based approach for cumulative incidence estimation of an emerging infectious disease that combines elements from several of the studies mentioned above. It requires repeated serosurveys (as in Buss et al. (23)) and makes use of antibody kinetic data (as in Williamson et al. (16)) to correct the estimates for seroreversion. In contrast to this empirical derivation of the distribution of times from seroconversion to seroreversion (similar to Prete et al. (24)), previous methods by Shioda et al. (19) and Buss et al. (23) assumed exponentially or Weibull-distributed seroreversion times. We validate the method for several in silico test cases and investigate the impact of various assumptions on the performance of the proposed method. We then apply the method to the Manaus data from Buss et al. and suggest that the cumulative incidence estimate of 76% in Manaus represents an overestimation.

## METHODS

Antibody waning is commonly observed after recovery from acute infections and can lead to seroreversion from a seropositive to a seronegative state over time (30, 31). To estimate cumulative incidence with correction for such seroreversion we propose a maximum-likelihood method that incorporates empirically derived probabilities of seroreversion. These can be derived from the dynamics of antibody decay.

### Distribution of seroreversion times

To derive empirically supported distributions of the times to seroreversion we need to make assumptions about the functional form of the antibody kinetics within infected individuals. Specifically, we assumed no delay between seroconversion (transition from seronegative to seropositive state) and peak antibody levels, both occurring a fixed duration $ {t}_{rec}$ after time of infection, and define an individual to be recovered at the time of seroconversion $ {t}_{rec}$. Furthermore, the term uninfected individual refers to any individual who has either not been infected or infected for less than time $ {t}_{rec}$, while the terms infected and recovered individuals are used interchangeably for those who were infected longer than time $ {t}_{rec}$ ago.

**Figure 1:**
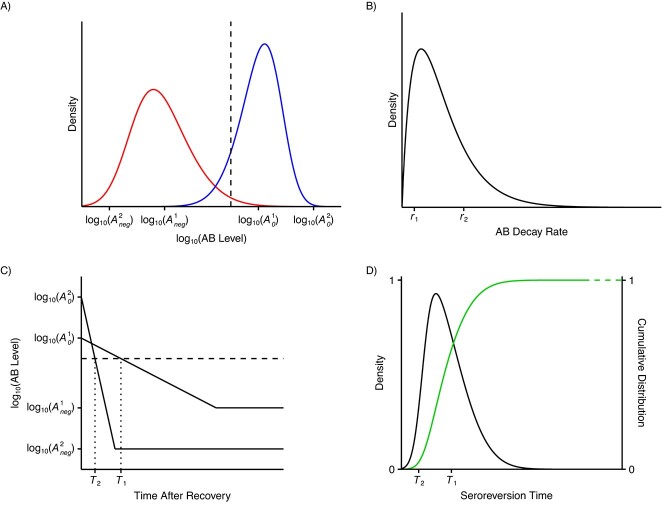
Schematic representation of how the distribution of seroreversion times is derived. A) Distributions of the decimal logarithms of antibody (AB) levels for positive controls (at peak, blue) and negative controls (red). The vertical line marks the cutoff for seropositivity. B) Distribution of antibody level decay rates for positive controls. C) Antibody dynamics exemplified for 2 individuals described by the tuples $\big({A}_0^1,{r}_1,{A}_{neg}^1\big)$ and $\big({A}_0^2,{r}_2,{A}_{neg}^2\big)$, where ${A}_0^i=A{(0)}^i$ and ${A}_{neg}^i$ are peak and background antibody levels drawn from the distributions in (A), and ${r}_i$ are antibody level decay rates drawn from the distribution in (B). The horizontal line marks the cutoff for seropositivity. D) Density (black) and cumulative distribution function (green) of seroreversion times approximated using $n$ tuples $(A(0),r,{A}_{neg})$ as described in i–iii (in Web Appendix 1).

The distribution of seroreversion times is derived from the distributions of quantitative antibody measures of positive controls at the time of peak antibody level, negative (before-pandemic) controls, and the distribution of antibody decay rates in positive controls under the assumption that antibodies decay exponentially after their peak. A schematic representation is shown in Figure 1 and details are given in Web Appendix 1 (available at https://doi.org/10.1093/aje/kwad226).

### Likelihood function for dichotomized data from sequential serosurveys

Maximizing the likelihood function that explains dichotomized (seropositive or seronegative) antibody data observed in a serosurvey is a common approach for seroprevalence estimation (32–35). The form of the likelihood and the number of optimized parameters depend on the available data and exact goal of the study. We derived a likelihood function that aims at explaining not only the results from a single serosurvey but a sequence of serosurveys conducted at regular intervals of 1 unit of time (e.g., 1 month or 1 week) starting shortly after the first recoveries in the population. We assumed that the individuals sampled at each time point represent independent draws from the population. We refer to such a sequence of serosurveys as 1 study. By integrating our control-data–derived knowledge on seroreversion time distribution, and consequently the probability to serorevert within $t$ units of time after recovery, ${p}_{rev}^{\mid pos, con}(t)$, and our knowledge on test sensitivity ($sens$) and specificity ($spec$), we correct for seroreversion of individuals due to antibody waning and for test accuracy. Let $Z$ be a ${\left\{0,1\right\}}^{\sum_{j=0}^m{n}_j}$ valued random variable describing the test results of all study participants in a study consisting of $m+1$ surveys with ${n}_j$ survey participants in survey $j$. Here, 0 represents a negative and 1 a positive serological test result. Then, the log-likelihood for the unknown per capita new infections between two surveys, ${r}_0,\dots, {r}_m$, given the data $Z=z$, is given by:

(1) \begin{align*} &\notag \log L\left({r}_0,\dots, {r}_m|Z=z\right)\\ {}& \quad =\sum_{j=0}^m\left\{{N}_j\!\times\! \log \left({p}_j\left({\Theta}_j\right)\right)\!+\!\left({n}_j\!-\!{N}_j\right)\!\times \!\log \left(1\!-\!{p}_j\left({\Theta}_j\right)\right)\right\}\!, \end{align*}

where the total number of survey participants, ${n}_j$, and the number of survey participants with positive test results, ${N}_j$, are extracted from the the data $z$, ${\Theta}_j$ is defined by $\big( sens, spec,{p}_{rev}^{\mid pos, con}\left(0+1/2\right),\dots, {p}_{rev}^{\mid pos, con}\big(j+1/2\big),{r}_0,\dots, {r}_j\big)$, and ${p}_j\left({\Theta}_j\right)$ is the probability that a randomly drawn individual in the population has a positive test result at the time of survey $j$. For a detailed derivation of the log-likelihood function and ${p}_j\left({\Theta}_j\right)$, see Web Appendix 1. The arguments ${r}_i^{\ast }$ which maximize the log-likelihood function in equation1 yield the cumulative incidence estimates ${c}_j^{\ast }={\sum}_{i=0}^j{r}_i^{\ast }$. For a detailed description of the optimization routine and on estimating confidence regions for the cumulative incidence estimates that account for uncertainties arising from both validation and study data, see Web Appendix 1.

### Simulations

#### Creating in silico studies—test cases.

Our method is tested using several in silico studies. Each in silico study consists of both validation and study data, with study data comprising data from a sequence of serosurveys. In brief, these data are sampled from the assumed true underlying distributions of peak level, background level and decay rate of antibodies (see Web Figure 1), as described in more detail in Web Appendix 1.

**Table 1:** Description of Test Cases, Hypothetical Epidemic Patterns for Which the Method Is Analyzed

**Test Case**	**No. of Monthly Surveys** ^**a**^	**No. of Epidemic Waves** ^**b**^	**Time of Wave, Month Past Outbreak** ^**b**^	**Total Fraction Infected During Each Wave, %**
1	9	1	3–4	40
2	9	1	6–7	40
3	9	2	3–4, 7–8	11, 22
4	9	2	3–4, 7–8	22, 11
5	15	2	3–5, 12–14	41, 25
6	9	0^c^		
7	17	0^c^		

^a^ In each survey 800–900 individuals are sampled from a population that is assumed to be large enough for these samples to have little overlap.

^b^ Any month in which more than 3% of the entire population is infected is considered to be (part of) an epidemic wave. Consecutive months each with more than 3% infected are defined as a single wave. Incidences in months that are not part of any epidemic wave range from 0% to 3%. An exact definition of the 7 test cases is given in equation 8 in Web Appendix 1.

^c^ Test cases 6 and 7 are characterized by consistently low monthly incidences of less than 2% of the population infected per month.

#### Performance analysis.

We defined the statistical power of this method as the proportion of in silico studies that estimate the cumulative incidence successfully at least 75% of all survey times within the study and semisuccessfully at the time of the latest survey within the study. Success is defined as a relative difference of less than 10% or an absolute difference of less than 2% between estimated and true cumulative incidence in addition to a difference of less than 2 standard deviations between estimated and true cumulative incidence. A semisuccess is reached if the relative difference between estimated and true cumulative incidence is less than 20% or the absolute difference is less than 3%. It is important to note that what we call power is different from the classical statistical power for hypothesis tests.

## RESULTS

### Theoretical results—method validation via in silico studies

**Figure 2:**
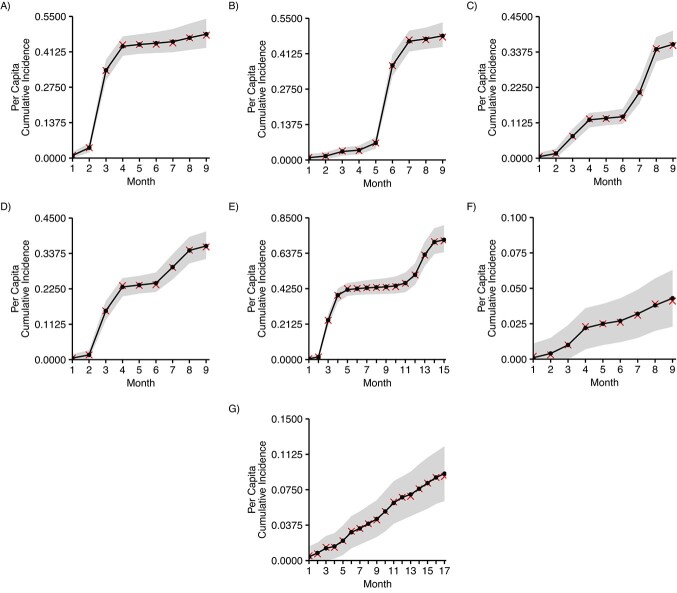
True cumulative incidences (red) and median of fitted cumulative incidences from ${N}_{sim}=10000$ in silico studies (black) with uniformly distributed recovery times between sequential surveys and a delay of 1 month between surveys for test cases 1–7 ((A) through (G), respectively). The shaded gray region is bounded by the 2.5% and 97.5% quantiles of the estimated cumulative incidences.

To validate the described method for cumulative incidence estimation from sequential serosurveys, we consider 7 test cases where an epidemic is observed for 9 months (in 5 test cases) and 15 and 17 months in 1 test case each. In these test cases, the true epidemic size ranges from consistently low, with monthly per capita incidences below 2%, to strong waves with up to 30% of the population infected within a single month. An overview summarizing the 7 test cases is given in Table 1. (For a detailed description see Web Appendix 1 and equation 8 therein.) In silico studies are created under the simplifying assumption that recoveries are uniformly distributed between any 2 consecutive surveys (alternative assumptions are discussed in Web Appendix 2 and Web Figures 2–5). Furthermore, the number of surveyed individuals in any survey of any in silico study is a randomly drawn integer between 800 and 900, the number of peak and background antibody levels in the validation data of any in silico study is set to 900, and the number of antibody decay rates in the validation data is fixed to 100. For each test case ${N}_{sim}=10,000$ in silico studies are conducted and cumulative incidences are estimated by maximizing the log-likelihood function in equation 1, with fixed parameters (sensitivity, specificity, and seroreversion probabilities) derived from the validation data of the respective in silico study. Figure 2 shows good agreement between true cumulative incidences and cumulative incidences fitted to data from the in silico studies, with powers ranging from 87.4% for test case 4 to 96.1% for test case 2 (see Web Table 1, row 1). The impact of variations in the delay between 2 consecutive surveys, in the correlation between peak antibody levels and antibody decay rates in the number of participants per survey, and in the assumed shape of the distribution of seroreversion times on the method’s performance are discussed in Web Appendix 2 and Web Figures 6–15. These analyses include several cases of mismatches between the model used for simulating and the model assumed when fitting the data—for example, different correlations or different distributions of seroreversion times (empirically derived for simulation vs. various others when fitting). Additionally, we compared the impact of a constant population followed throughout the various surveys within 1 study instead of disjoint survey populations.

**Figure 3:**
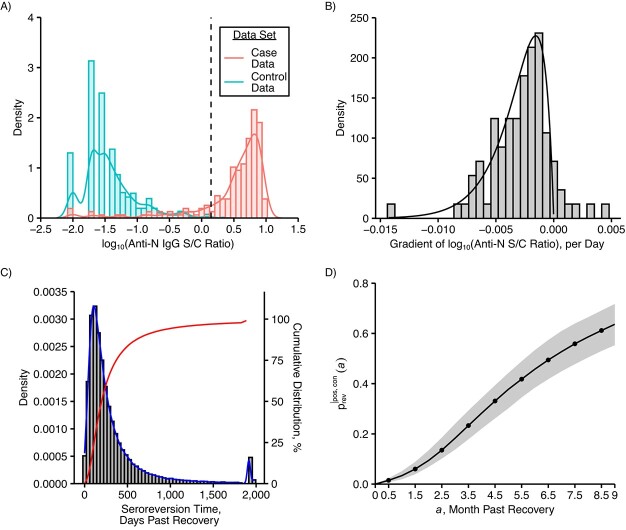
A) Histogram of decimal logarithms of anti–nucleocapsid protein (anti-N) immunoglobulin G (IgG) signal-to-cutoff (S/C) ratios for case and control data (from Buss et al. (23)) with the densities of the respective smoothed empirical distributions. The vertical line marks the cutoff for seropositivity. B) Histogram of the anti-N IgG decay rates. More specifically, the histogram of the gradients of the decimal logarithm of anti-N IgG S/C ratios in convalescent controls (from Buss et al. (23)) with the density of the gamma distribution that was fitted to the absolute values of the negative gradients. C) Histogram, probability density function ${f}_{T_{\Theta}}$ (blue) and cumulative distribution function ${F}_{T_{\Theta}}$ (red) for seroreversion times ${T}_{\Theta}$ of initially seroconverted individuals. For better visibility we combined all seroreversion times larger than 5 years and display them at 5 years plus 90 days. Furthermore, we combined all individuals that never serorevert and display them at 5 years plus 150 days. D) Probabilities to serorevert within a given time past recovery. Shaded region is bounded by the 2.5 and 97.5% quantiles obtained when resampling the validation data and recalculating the seroreversion probabilities 100 times.

### A real world example—estimating the spread of SARS-CoV-2 in Manaus from March to October 2020

In their paper on the attack rate of SARS-CoV-2, which corresponds to what we call cumulative incidence, in the Brazilian Amazon, Buss et al. (23) used data on anti–nucleocapsid protein (anti-N) immunoglobulin G (IgG) levels (signal-to-cutoff (S/C) ratios) from repetitive (monthly from March to October 2020) but independent serosurveys in Manaus to estimate the monthly cumulative incidence. Here, independence means that different cohorts comprising 800–900 individuals each, sampled from the same population (blood donors in Manaus), were surveyed at each time point. In addition to the serosurvey data, Buss et al. reported anti-N IgG levels of convalescents 20–50 days past symptom onset (positive controls), anti-N IgG gradients in convalescents, and anti-N IgG levels of before-pandemic controls (negative controls). A detailed description of these validation data can be found in Web Appendix 1.

As anti-N IgG is estimated to peak approximately 3–4weeks after symptom onset, we assumed that the levels from the positive control group were a good representation of peak IgG levels (12). Consequently, when deriving the distribution of seroreversion times as described in i–iii in Web Appendix 1, and schematically represented in Figure 1, we assumed that peak and background antibody levels are distributed according to the empirical distributions of the positive and the negative control groups, respectively (see Figure 3A). Meanwhile, since we assumed antibody levels are decreasing, we ignored positive gradients (7/88) and fitted a gamma distribution to the absolute values of the negative gradients (81/88; see Figure 3B). The derived distribution of seroreversion times is shown in Figure 3C. Since serosurveys are performed monthly, we extracted the probabilities to serorevert within the first 0.5–8.5 months after peak antibody titer (see Figure 3D, black curve). We estimated that within 6 months past seroconversion, approximately 46% (38–53%) serorevert. This is roughly in accordance with the results reported in Krutikov et al. (17) but lower than the 81% found in Buss et al. (23) (see Web Figure 16A for a comparison of seroreversion times derived using the empirical approach described in this article (red curve) and those derived by Buss et al. (green curve)). Buss et al. did not fit the seroreversion rate to longitudinal antibody data but rather derived it as a side product when estimating monthly cumulative incidence from serosurvey data.

**Figure 4:**
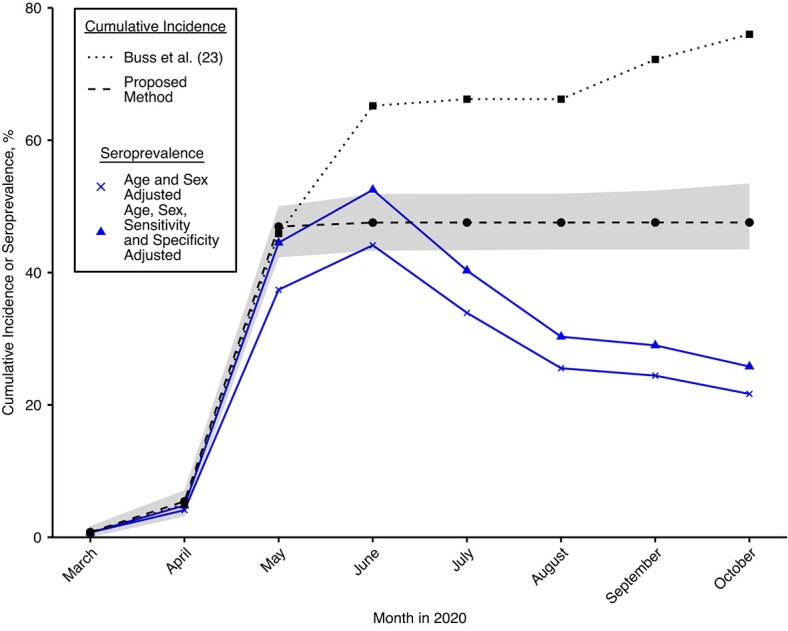
Cumulative incidence estimates applying our method (black dots) to age- and sex-adjusted observed seroprevalences reported by Buss et al. (23) (blue crosses). The shaded region is the bootstrapped 95% confidence region using 1,000 bootstrap samples. For comparison, seroprevalence observations additionally adjusted for test sensitivity and specificity (blue triangles) and Buss et al.’s cumulative incidence estimates (black squares) (23) are shown.

When correcting the cumulative incidence estimate for seroreversion using the empirically supported profile, we obtained a cumulative incidence estimate of 47.6% (bootstrap 95% confidence region (CR): 43.5, 53.5) in October 2020—roughly 30 percentage points lower than Buss et al.’s estimate of 76.0% and outside the 95% confidence interval (66.6, 97.9), that is, significantly lower (see Figure 4, dashed black curve, and Table 2). The cumulative incidence increased from 0.8% (95% CR: 0, 1.7) in March 2020 to 47% (95% CR: 42.3, 50.0) in May 2020 and has been almost constant from May through October 2020, with minimal nonsignificant increases in June. These results are consistent with the estimated cumulative incidence of 41.53%–44.82% in Manaus 6 months after the start of the epidemic determined in the DETECTCoV-19 cohort study (36). Our cumulative incidence estimate in June is significantly lower than Buss et al.’s age-, sex-, sensitivity- and specificity-adjusted estimate. This suggests that the raw seroprevalence estimates observed in June may be high, based on some bias introduced by the convenience sample of blood donors. The selection bias hypothesis is further supported by reestimating cumulative incidences when dropping the data from the June survey. Compared with using data from all surveys (including June), this reduced the cumulative incidence estimates from May through October (reductions not significant, see Web Figure 17). By contrast, dropping the data from either the May or July surveys rendered the estimates almost unchanged (except for wider confidence intervals due to reduced number of samples).

## DISCUSSION

In this study, we have proposed an empirically supported method for seroreversion correction in serosurveys. The method can be used in any situation where a disease enters a previously naive population, individuals are protected from reinfection for the duration of the study, and antibodies against the new antigen wane exponentially in recovered individuals. It also requires the availability of a quantitative serological assay for antibodies against the new antigen, as well as validation data from individuals with known past infection and from individuals prior to the introduction of the disease in the population. This empirical approach to seroreversion correction is based on the distribution of seroreversion times after recovery, the estimation of which requires peak antibody levels and decay rates (longitudinal data) of positive controls.

Considering 7 different test scenarios, simulating studies consisting of repeated serosurveys under various assumed incidence curves, has shown that, in general, the method successfully approximates the cumulative incidences at the times of the surveys. At times when the disease incidence increases, the method overcorrects for seroreversion, while it undercorrects when disease incidence decreases (see Web Appendix 2). The strength of under- or overcorrection depends on the rate of antibody waning and the delay between consecutive surveys. Thus, if antibody waning is fast, or disease incidence changes rapidly, frequent sampling is required (see Web Appendix 2). The method can be improved in the future by allowing the integration of information on the general shape of the incidence curve during the time of the study.

**Table 2:** Cumulative Incidence Estimates (%) Using Empirically Derived Seroreversion Times and Age- and Sex-Adjusted Seroprevalence Estimates

**Month in 2020**	**Age- and Sex-Adjusted Seroprevalence Estimate^**a**^**	**Cumulative Incidence**	**95% Confidence Region**
March	0.72	0.77	0.00, 1.69
April	4.10	5.42	3.18, 7.10
May	37.40	46.95	42.29, 50.01
June	44.13	47.55	43.31, 51.86
July	33.91	47.56	43.39, 51.90
August	25.54	47.56	43.50, 51.91
September	24.42	47.57	43.50, 52.38
October	21.66	47.57	43.52, 53.46

^a^ Reported by Buss et al. (23).

For all test scenarios maximal power has been reached when sampling 10^3.5^ or more individuals per survey (see Web Appendix 2). Lower sample sizes resulted in reduced power, while sampling more individuals did not increase the method’s power. These results were based on the assumption that the cohorts tested in the different surveys within one study are disjoint. If instead a constant cohort was followed over time, the proposed method in general still succeeded at estimating the monthly cumulative incidences for large enough cohort sizes. However, the size of a constant cohort required to reach the same power as when using disjoint cohorts needed to be greater than the sizes of the disjoint cohorts (see Web Figure 13). The reason for this is that, once infected, future antibody levels are predetermined (by peak and decay rate of the individual’s antibody level). Hence the number of survey participants that yield new information reduces with every survey.

Often seroreversion is not corrected for in serosurveys, which, in the context of antibodies that decay relatively fast, can lead to significant underestimation of cumulative incidences. If we had ignored correction for seroreversion by setting the corresponding probabilities to zero (see Web Appendix 2), we would have failed to correctly estimate cumulative incidence in all test cases with the exception of test case 6 (see Web Table 2). Two previous methods for seroreversion correction in the context of sequential serosurveys, introduced by Buss et al. (23) and Shioda et al. (19), assumed exponentially distributed seroreversion times (times from seroconversion to seroreversion) (23) or Weibull-distributed seroreversion times (19). Due to identifiability reasons, Shioda et al. assumed a fixed standard deviation of 50 days for this Weibull-distribution. By simulating study data using empirical data on antibody kinetics and using seroreversion probabilities derived under the assumptions that seroreversion times are exponentially or Weibull-distributed in the model used for fitting the simulated data (model mismatch), we have shown that these previous assumptions are in conflict with empirical data on antibody kinetics (see Web Appendix 2). Previous studies have reported a correlation between peak antibody levels and antibody decay rates after, for example, SARS-CoV-2 infection. We have shown that if the validation data approximately mirrors the true underlying correlation, our method, in general, performs well at estimating the cumulative incidence irrespective of the strength of the correlation (see Web Appendix 2). In some cases the method even predicted the cumulative incidence accurately if the correlation in the validation data and the true correlation did not match. However this was not true for all test scenarios.

The presented method can be applied to stratified data (e.g., age-stratified) by estimating cumulative incidences for each subpopulation individually and, if needed, combining the stratified estimates into a weighted population average. If not only the cumulative incidences but also the antibody dynamics vary between subpopulation (as might be the case for young vs. elderly), then stratified validation data is required, and seroreversion probabilities need to be estimated separately for each subpopulation.

Many infectious diseases do not confer perfect immunity after infection. In situations where: 1) the aim of a study is to—at the time of each survey—estimate the fraction of individuals who have been infected at least once, and 2) antibody dynamics after reinfection resemble antibody dynamics after primary infection (in terms of peak, decay, and background level), the method can be adapted to account for reinfections. To this end, the fractions of individuals who had recovered for a given amount of time (say $a$ units) at the time of a given survey needs to be replaced by the fractions of individuals whose latest recovery had been $a$ units of time before the given survey. The precise definition of this depends on whether, for how long, and at what level infection confers immunity and is outside the scope of this article.

Recently, it has been shown that if quantitative antibody measurements are available, cutoff-free methods that avoid dichotomizing study participants into antibody-positive or antibody-negative are beneficial compared with cutoff-based methods when estimating cumulative incidence from a single serological survey (34, 37). In the future, we plan to adapt the approach presented in this article and introduce a similar cutoff-free approach for cumulative incidence estimation from sequential cross-sectional serological surveys.

Applying our method to serosurvey data from Manaus (23) suggested that the previously reported cumulative incidence estimate of 76% by October 2020 is a significant overestimation. We predicted a cumulative incidence approximately 30 percentage points lower, at 47.6% (43.5% to 53.5%), which is in line with Lalwani et al. (36). Similar to Buss et al., we estimated the cumulative incidence under the assumption that incidence can only increase. The observed seroprevalences show large drops from May to June to July. To explain this, Buss et al. predicted large proportions of seroconverted individuals to serorevert within the first or second month past seroconversion. This however, is not in accordance with the anti-N IgG dynamics observed in the convalescent control group (see Web Figure 16A, green vs. red curves). By contrast, our method based the seroreversion correction on the antibody dynamics observed in convalescent plasma donors and predicted cumulative incidence estimates in June significantly below the respective sensitivity and specificity adjusted seroprevalence estimates, suggesting a possible selection bias in the data. This hypothesis of selection bias in the June serosurvey was also supported by reestimation of cumulative incidences when ignoring data from a single serosurvey. While ignoring the data from May or July left the estimates almost unchanged, they were reduced when ignoring the June serosurvey. The data from convalescent individuals showed some evidence for a correlation between anti-N IgG peak and decay rate. Accounting for this in the seroreversion probabilities (see Web Figure 18A), however, did not result in significantly different cumulative incidence estimates (see Web Appendix 2 and Web Figure 18B). The distributions of positive control peak antibody levels and decay rates and the distribution of negative control antibody levels used to derive the in silico studies in the test scenarios were chosen to be very similar to those observed in the validation data from Manaus. When each survey consisted of 800–900 individuals, our method displayed a relatively high power of more than 75% for all test scenarios under the assumption of disjoint cohorts at each survey within 1 study. It is not clear to us whether this is guaranteed in the Manaus data set or whether there is some overlap of the cohorts. However, since the predicted powers of the proposed method still ranged above 67% even if a constant cohort was followed through time (see Web Figure 13), these results justify a certain degree of trust in our cumulative incidence estimates for Manaus.

As a byproduct, this method returned the distribution of seroreversion times estimated from positive controls’ dynamics. From this distribution one could derive the fraction of individuals that have seroreverted at a given time past recovery. We have predicted from the Manaus data set that, for anti-N using the Abbott (Abbott Park, Illinois) Architect SARS-CoV-2 IgG assay, 46% of seroconverters serorevert within the first 6 months past seroconversion, lower than the 81% found by Buss et al. (23) but roughly in accordance with Krutikov et al. (17).

The positive control data from Manaus that was used to derive the seroreversion probability is only representative of symptomatic, nonhospitalized COVID-19 cases. However, antibody levels and therefore time to seroreversion vary with disease severity (38). In Web Appendix 2 we compared the fitted cumulative incidences with those obtained when using a set of positive controls that is closer to the survey data in terms of disease severity. We found that, while estimated cumulative incidences are slightly larger, due to faster seroreversion derived from the alternative positive control group, the estimated cumulative incidence in October 2020 is still significantly below that estimated by Buss et al.

Even though the alternative, empirically supported seroreversion correction does not provide a full explanation for the unexpected resurgence of the SARS-CoV-2 epidemic in Manaus, it contributes to solving the puzzle by providing a lower estimate of the cumulative incidence. Beyond its relevance for the Brazilian serosurvey, the approach to adjusting for seroreversion presented here provides an important, empirically supported method that could be used in any serosurvey in which the antibody levels wane over time.

## Supplementary Material

Web_Material_kwad226
