# Supplementary material for: Correcting for Antibody Waning in Cumulative Incidence Estimation From Sequential Serosurveys
Source: Am J Epidemiol. 2023 Nov 27;193(5):777–86. doi: 10.1093/aje/kwad226 (PMC11074712; doi:10.1093/aje/kwad226)
Supplement: Web_Material_kwad226 [file web_material_kwad226.pdf]

Correcting for antibody waning in cumulative incidence estimation from sequential serosurveys

Sarah Kadelka, Judith A Bouman, Peter Ashcroft, Roland R Regoes

Contents

Web Appendix 1 – Methods **2**
Distribution of sero-reversion times . . . 2
Likelihood function for dichotomized data from sequential serosurveys . . . 2
Confidence region . . . 4
Simulations . . . 4
Creating *in silico* studies – Test cases . . . 4
Test scenarios . . . 5
Real world data shared by Buss *et al.* . . . 5
Optimization routine . . . 6
Codes and data . . . 6

Web Appendix 2 – Results **6**
Theoretical results: Sensitivity analysis . . . 6
Distribution of recoveries between consecutive surveys . . . 6
Time between surveys . . . 6
Correlated antibody peak and decay . . . 7
Number of individuals per survey . . . 7
Varying probabilities of sero-reversion . . . 7
Real world example . . . 8
Correlation between peak and decay rate of antibody level . . . 8
Different control data . . . 9

Web Tables **10**
Web Table 1 . . . 10
Web Table 2 . . . 10

Web Figures **11**
Web Figure 1 . . . 11
Web Figure 2 . . . 12
Web Figure 3 . . . 13
Web Figure 4 . . . 14
Web Figure 5 . . . 15
Web Figure 6 . . . 16
Web Figure 7 . . . 17
Web Figure 8 . . . 18
Web Figure 9 . . . 19
Web Figure 10 . . . 20
Web Figure 11 . . . 21
Web Figure 12 . . . 22
Web Figure 13 . . . 23
Web Figure 14 . . . 24
Web Figure 15 . . . 25
Web Figure 16 . . . 26
Web Figure 17 . . . 26
Web Figure 18 . . . 27

Web Appendix 1: Methods

Distribution of sero-reversion times

Antibody waning is commonly observed after recovery from acute infections and can lead to sero-reversion from a sero-positive to a sero-negative state over time (1; 2). The time from recovery to sero-reversion depends on several factors: antibody level at recovery, antibody decay pattern and rate and the assay-specific sero-positivity threshold. In the following we make the simplifying assumption of a rapid incline of antibodies at the time of recovery, i.e. we assume no delay between sero-conversion and peak antibody levels and define recovery as the time of sero-conversion (i.e. the time of peak antibody level). To derive the distribution of sero-reversion times we use:

(a) The distribution of quantitative antibody measures of positive controls at the time of peak antibody level (as exemplified in Figure 1A);

(b) The distribution of antibody decay rates in positive controls (as exemplified in Figure 1B);

(c) The distribution of quantitative antibody measures of negative (pre-pandemic) controls (as exemplified in Figure 1A);

(d) The sero-positivity threshold, Θ, of the assay used for antibody quantification in (a)-(c) (as exemplified in Figure 1A).

For the antibody dynamics, we assume that antibody levels decrease exponentially from their peak level after recovery until reaching the background level observed in negative controls. Concretely, if we define $A(t)$ as the antibody level at t days after recovery (=time of peak antibody level), then this satisfies

$$A(t) = \max\left[A(0) \cdot 10^{-rt}, A_{\text{neg}}\right]. \tag{1}$$

From the distributions in (a)-(c) and the antibody dynamics in (1) the distribution of sero-reversion times is approximated in the following way:

i) A large number n of tuples $(A(0), r, A_{neg})$ is sampled from the distributions in (a)-(c) (each parameter is drawn independently from its own distribution);

ii) For each tuple $(A(0), r, A_{neg})$ the predicted trajectory of the antibody level, $A(t)$ is computed, as exemplified in Figure 1C;

iii) For each trajectory that starts above the sero-positivity threshold Θ, the time T_Θ at which $A(T_\Theta) = \Theta$ is computed, i.e. the time after recovery at which the antibody level crosses the threshold and the individual sero-reverts. From these values, the empirical density and cumulative distribution functions f_{T_Θ} and F_{T_Θ} of the sero-reversion time T_Θ are derived, as exemplified in Figure 1D.

Following SARS-CoV-2 infections, several studies have presented evidence for a correlation between $A(0)$ and r (3; 4). To incorporate such correlation in our method, one extra step is added to constructing the distribution of sero-reversion times described in (i)–(iii):

i_b) The n independently drawn entries of $A(0)$ and r are rearranged to mimic the Spearman correlation coefficient observed in the validation data.

Changes in the correlation between peak antibody level and decay rate result in changes of the distribution of sero-reversion times. In a scenario where antibody peaks and decay rates are perfectly positively correlated, i.e antibodies decay fastest in those individuals with the highest peak antibody levels, the distribution of sero-reversion times is narrow, while it is widest for a perfect negative correlation between antibody peak and decay rate. In general, how a change in correlation influences the distribution of sero-reversion times depends on several factors: the values of peak and decay rate, and the positivity threshold.

Likelihood function for dichotomized data from sequential serosurveys

We derive a likelihood function that can be used to obtain longitudinal cumulative incidence estimates from a sequence of serosurveys for an emerging infectious disease. In these serosurveys independent samples of the same population are serologically tested for the presence of antibodies specific to the pathogen of interest. By integrating our knowledge on antibody kinetics, we correct for antibody-waning-induced sero-reversion of individuals. Optimization of parameters to maximize the likelihood function results in cumulative incidence estimates at the time of each survey.

Ignoring any inter-individual heterogeneity, we assume a fixed duration t_{rec} from infection to recovery. As in the previous section recovery is assumed to coincide with peak antibody levels and, for individuals whose peak is above the positivity threshold, with seroconversion. The term *uninfected individual* refers to any individual who has either not been infected or infected for less than t_{rec} days, while the terms *infected* and *recovered individuals* are used interchangeably for those infected more than t_{rec} days ago. Serosurveys of a population are performed at regular time intervals (e.g. monthly or biweekly). The duration between two sequential surveys is set as the unit of time t, and $t = 0$ refers to the time of the first survey, which we assume to take place shortly after the first recoveries in the population, *i.e.* we assume that there is no history of the given disease in the population long before the first survey.

The per capita new recoveries at time $t = i$, r_i, for $i \in \{0, 1, \dots, m\}$ describe the fraction of the population that recovered between survey $i-1$ and i for $i > 0$ and before the first survey for $i = 0$. Under the assumption that during the study period individuals are protected from reinfection, the cumulative incidence at time $T \in \{0, 1, \dots, m\}$ is then given by $c_T = \sum_{i=0}^{T} r_i$. Assuming constant antibody levels in the absence of infection, the fraction of seropositives among uninfected individuals can be approximated by one minus the test's specificity $(1 - spec)$. If case sera in the validation set represent peak antibody levels, the fraction of at recovery seroconverted recovered individuals can be approximated by the test's sensitivity ($sens$). Lastly, let $f_{T_\Theta}(t)$ be the probability density function of T_Θ (see figure 1D), then the fraction of at recovery seroconverted individuals that serorevert within t units of time after recovery is given by

$$p_{rev}^{|pos,con}(t) = F_{T_\Theta}(t),$$

where F_{T_Θ} is the corresponding cumulative distribution function. Let X_i^j be $\{0, 1\}$-valued random variables describing the test results of individuals $i = 1, \dots, n_j$ at time $j = 0, \dots, m$ with 1 indicating a positive and 0 a negative test result. Then, assuming no individuals are sampled twice, all X_i^j are independent and

$$X_i^j \sim Bin(1, p_j(\boldsymbol{\Theta}_j)) = Bin\big(1, p_{T_{max}=j}(\boldsymbol{\Theta}_{T_{max}=j})\big), \tag{2}$$

where

$$p_{T_{max}}(\boldsymbol{\Theta}_{T_{max}}) = \underbrace{\left(1 - \sum_{i=0}^{T_{max}} r_i\right) \times (1 - spec)}_{(I)} + \underbrace{\sum_{a=0}^{T_{max}} \left\{ r_{T_{max}-a} \times sens \times (1 - p_{rev}^{|pos,con}(a + 1/2)) \right\}}_{(II)}, \tag{3}$$

and

$$\boldsymbol{\Theta}_{T_{max}} = \Big(sens, spec, p_{rev}^{|pos,con}(0 + 1/2), \dots, p_{rev}^{|pos,con}(T_{max} + 1/2), r_0, \dots, r_{T_{max}}\Big). \tag{4}$$

In brief, this means that the probability for a randomly selected individual in the $(T_{max}+1)^{th}$ serosurvey, performed at time T_{max}, to have a positive test result is the sum of (I) the probability that the selected individual has not recovered from infection by time T_{max} but has an antibody level above the positivity threshold, and (II) the sum over $0 \le a \le T_{max}$ of the probabilities that the selected individual recovered $a + 1/2$ units of time before T_{max}, had seroconverted at its recovery and has not sero-reverted in the $a + 1/2$ units of time since recovery. The assumption that the first survey is conducted shortly after the emergence of the epidemic explains why only the past T_{max} units of time need to be considered. Furthermore, without any knowledge on the temporal distribution of recoveries between two surveys the most general assumption is that recoveries occur at the midpoint between two surveys which explains the added $1/2$ units of time in the sero-reversion probability term. For example, individuals included in the fraction of the population, r_j, that recovered between surveys $j - 1$ and j have, on average, already been recovered for $1/2$ units of time at the time of survey j. Thus, by the time of survey j they have sero-reverted with a probability $p_{rev}^{|pos,con}(0 + 1/2)$. The independence of the X_i^j and $\boldsymbol{\Theta}_0 \subseteq \boldsymbol{\Theta}_1 \subseteq \ldots \subseteq \boldsymbol{\Theta}_m$, implies for the random vector $Z = (X_1^0, \dots, X_{n_0}^0, \dots, X_1^m, \dots, X_{n_m}^m)$, that

$$\begin{aligned} P(\boldsymbol{Z} = \boldsymbol{z}|\boldsymbol{\Theta}_m) &= P(X_1^0 = x_1^0, \dots, X_{n_0}^0 = x_{n_0}^0, \dots, X_1^m = x_1^m, \dots, X_{n_m}^m = x_{n_m}^m|\boldsymbol{\Theta}_m) \\ &= \prod_{j=0}^{m} \prod_{i=1}^{n_j} \left\{ (p_j(\boldsymbol{\Theta}_j))^{x_i^j} (1 - p_j(\boldsymbol{\Theta}_j))^{1-x_i^j} \right\}. \end{aligned} \tag{5}$$

For fixed parameters $sens, spec, p_{rev}^{|pos,con}(0 + 1/2), \dots, p_{rev}^{|pos,con}(m + 1/2)$ the likelihood function of $r_0, \dots, r_m$ given the data $\boldsymbol{Z} = \boldsymbol{z}$ is given by

$$L(r_0, \dots, r_m|\boldsymbol{Z} = \boldsymbol{z}) = \prod_{j=0}^{m} \prod_{i=1}^{n_j} \left\{ (p_j(\boldsymbol{\Theta}_j))^{x_i^j} (1 - p_j(\boldsymbol{\Theta}_j))^{1-x_i^j} \right\}, \tag{6}$$

and consequently the log-likelihood function is given by

$$\begin{aligned}
&\log L(r_0, \dots, r_m | \boldsymbol{Z} = \boldsymbol{z}) \\
&= \sum_{j=0}^{m} \sum_{i=1}^{n_j} \log \left\{ (p_j(\boldsymbol{\Theta}_j))^{x_i^j} (1 - p_j(\boldsymbol{\Theta}_j))^{1-x_i^j} \right\} \\
&= \sum_{j=0}^{m} \sum_{i=1}^{n_j} \left\{ x_i^j \log(p_j(\boldsymbol{\Theta}_j)) + (1 - x_i^j) \log(1 - p_j(\boldsymbol{\Theta}_j)) \right\} \\
&= \sum_{j=0}^{m} \left\{ N_j \times \log(p_j(\boldsymbol{\Theta}_j)) + (n_j - N_j) \times \log(1 - p_j(\boldsymbol{\Theta}_j)) \right\},
\end{aligned} \tag{7}$$

where $N_j = \sum_{i=1}^{n_j} x_i^j$ equals the total number of participants in survey j that are tested positive.
The optimal parameters $r_0^*, \dots, r_m^*$ are obtained by maximizing the log-likelihood function in equation (7) and yield the cumulative incidence estimates $c_j^* = \sum_{i=0}^{j} r_i^*$.

Confidence region

To estimate a confidence region for the cumulative incidences, we suggest a bootstrapping algorithm that accounts for uncertainties arising from the lack of accuracy of both validation and study data. In a first step, validation data (peak, background and decay rate) are resampled and test accuracy (sensitivity and specificity) as well as sero-reversion probabilities re-evaluated. In a second step, study data are resampled and the log-likelihood with fixed parameters from step one is maximized for the resampled study data from step two.

The log-likelihood function described in equation (7) depends only on the numbers of positive and negative test results, but not their ordering. Therefore, to create bootstrap samples of the study data, it suffices to resample the numbers of positive samples $N_j^{boot_i}$ in the surveys of n_j probands at time $j = 0, \dots, m$. Thus, $N_j^{boot_i}$, $i = 1, \dots, N_{boot}$, are realization of $Bin\left(n_j, \frac{N_j}{n_j}\right)$-distributed random variables. For each bootstrap sample $(N_0^{boot_i}, \dots, N_m^{boot_i})$, $i = 1, \dots, N_{boot}$, maximizing the log-likelihood function yields parameter estimates $r_0^i, \dots, r_m^i$ and consequently cumulative incidence estimates $c_0^i, \dots, c_m^i$, where $c_j^i = \sum_{k=0}^{j} r_k^i$. A 95% confidence region $I \subset \mathbb{R}^{m+1}$ in which all entries of $\boldsymbol{c} = (c_0, \dots, c_m)$ fall simultaneously with confidence 95% is given by $I = I_{c_0} \times \dots \times I_{c_m}$, where I_{c_j} is the interval bounded by the $0.025/(m+1)$ and $1 - 0.025/(m+1)$ percentiles of the N_{boot} bootstrapped estimates for c_j, $j = 0, \dots, m$.

Simulations

Creating *in silico* studies – Test cases

To test our method, we create *in silico* studies. We assume that the decimal logarithms of quantitative antibody peak and background values are skew normally distributed with location $\xi_{peak} = 0.9$, scale $\omega_{peak} = 0.54$, shape $\alpha_{peak} = -8$ and location $\xi_{background} = -1.8$, scale $\omega_{background} = 0.75$, shape $\alpha_{background} = 2$. Furthermore, we assume that the decimal logarithm of quantitative antibody levels decays linearly with decay rates being gamma distributed with shape $\alpha_{decay} = 1.93$ and rate $\beta_{decay} = 600.52$. The distributions are shown in Web Figure 1.

In silico studies are defined by two data sets. First, the validation data consisting of N_{peak} and $N_{background}$ peak and background antibody level measurements in combination with N_{decay} daily antibody decay rates, sampled from the respective distributions. Second, the study data itself, which, for true underlying per capita new infections $R_0, \dots, R_{M-1}$, is created as follows: For each of the M serosurveys, the number of virtual individuals included in the survey is randomly sampled from $\mathcal{U}\{N_{min}, N_{max}\}$, where N_{min} and N_{max} are pre-defined upper and lower bounds for the number of individuals per survey. For each virtual individual in a given survey $m \in \{0, \dots, M-1\}$, we randomly sample whether the individual recovered and, if so, the time at which the individual recovered by drawing from $\{0, \dots, M-1, \text{not recovered}\}$ with corresponding probabilities of success $\{R_0, \dots, R_m, \vec{0}_{(M-1)-m}, 1 - \sum_{k=0}^{m} R_k\}$. For each virtual individual a peak, background and decay rate of antibody level are drawn from the respective distribution. If the virtual individual is not recovered then its antibody level is set to the sampled background level. Otherwise, if the virtual individual stems from a survey at time m and has recovered at time $0 \leq j \leq m$, then its antibody level is set to

$$\max \left\{ peak - (fac \times (m - j) + t_1) \times decay,\ background \right\},$$

where t_1 is uniformly distributed on $\{0, .., fac-1\}$, *i.e.* the probability mass function of t_1 is given by $f_{t_1}(t) = 1/fac$ if $t \in \{0, \dots, fac-1\}$ and $f_{t_1}(t) = 0$ otherwise. The factor $fac = $ (unit of time)/day is included since the decay rate is sampled from the distribution of daily decay rates.

We consider four alternatives to the uniform distribution of recoveries between two surveys: (1) all recoveries at the day after the former study ($f_{t_1}(t) = 1$ if $t = fac - 1$ and $f_{t_1}(t) = 0$ otherwise), (2) daily recoveries decay exponentially between two studies ($f_{t_1}(t) = \exp(-g(fac - t))/\sum_{t=0}^{fac-1} \exp(-g(fac - t))$ if $t \in \{0, \ldots, fac - 1\}$ and $f_{t_1}(t) = 0$ otherwise, with g restricted to $[\ln(2)/300, \ln(2)/5]$), (3) daily recoveries grow exponentially between two studies ($f_{t_1}(t) = \exp(g(fac - t))/\sum_{t=0}^{fac-1} \exp(g(fac - t))$ if $t \in \{0, \ldots, fac - 1\}$ and $f_{t_1}(t) = 0$ otherwise, $g \in [\ln(2)/300, \ln(2)/5]$) and (4) all recoveries at the day of the latter study ($f_{t_1}(t) = 1$ if $t = 0$ and $f_{t_1}(t) = 0$ otherwise). We assume that the form of the distribution is the same on all intervals between any two consecutive surveys. However if (2) or (3) are assumed the value for g is allowed to vary.

If a Spearman correlation of strength ρ is assumed between peak and decay rate an additional step is added before calculating the antibody level of study participants: Peaks and decay rates from all study participants are rearranged to mimic the assumed Spearman correlation ρ and afterwards the pairs (peak, decay rate) are randomly redistributed among the study participants.

Test scenarios

The following seven test case scenarios are considered: First, a scenario where the first nine months of an epidemic are taken into account with one strong wave of infections occurring during months 3 to 4, 40% of the total population infected in those two months and lower incidences in all other months. Second, a strong wave with 40% of the population infected during months 6-7 and low incidences for months 1-5 and 8-9. Third, two waves with 11% and 22% of the population infected during months 3-4 and 7-9, respectively, and low incidences in the remaining four months. Fourth, two waves with 22% and 11% of the population infected during months 3-4 and 7-8, respectively, and low incidences in the remaining five months. Fifth, a scenario of two waves with 41% and 25% infected during months 3-5 and 12-14, low incidences in the remaining months and a total observation time of 15 months. Lastly, the sixth and seventh scenarios consist of consistently low incidences for 9 and 17 months, respectively. Specifically, the following true values of monthly per capita new recoveries are assumed

$$\begin{aligned}
\boldsymbol{r}_1 &= (1, 3, 30, 10, 0.1, 0.1, 0.5, 2, 1)\% \\
\boldsymbol{r}_2 &= (1, 0.5, 2, 0.1, 3, 30, 10, 0.1, 1)\% \\
\boldsymbol{r}_3 &= (0.5, 1, 5.5, 5.5, 0.1, 0.2, 8, 14, 1)\% \\
\boldsymbol{r}_4 &= (0.5, 1, 14, 8, 0.1, 0.2, 5.5, 5.5, 1)\% \\
\boldsymbol{r}_5 &= (0.5, 1, 22, 15, 4, 0.1, 0.5, 0.1, 0.1, 0.4, 2, 5, 12, 8, 0.5, 0.5)\% \\
\boldsymbol{r}_6 &= (0.2, 0.1, 0.7, 1.3, 0.2, 0.1, 0.5, 0.8, 0.2)\% \\
\boldsymbol{r}_7 &= (0.5, 0.2, 0.7, 0.02, 0.7, 1, 0.3, 0.5, 0.4, 0.9, 1, 0.5, 0.1, 0.8, 0.6, 0.6, 0.07)\%
\end{aligned} \tag{8}$$

To investigate the impact of the duration between two surveys on the performance of the proposed method, the same seven test cases with surveys performed in the first month and then followed up every 2 months are considered. The assumed true values of per capita new recoveries are summarized in equation (9).

$$\begin{aligned}
\boldsymbol{r}_1 &= (1, 33, 10.1, 0.6, 3)\% \\
\boldsymbol{r}_2 &= (1, 2.5, 3.1, 40, 1.1)\% \\
\boldsymbol{r}_3 &= (0.5, 6.5, 5.6, 8.2, 15)\% \\
\boldsymbol{r}_4 &= (0.5, 15, 8.1, 5.7, 6.5)\% \\
\boldsymbol{r}_5 &= (0.5, 17, 4.1, 0.6, 0.5, 7, 20, 1)\% \\
\boldsymbol{r}_6 &= (0.2, 0.8, 1.5, 0.6, 1)\% \\
\boldsymbol{r}_7 &= (0.5, 0.9, 0.72, 1.3, 0.9, 1.9, 0.6, 1.4, 0.67)\%
\end{aligned} \tag{9}$$

Real world data shared by Buss *et al.*

In their paper on the attack rate of SARS-CoV-2 in the Brazilian Amazon (5), the authors reported and comprehensively shared the following antibody data which were determined using the Abbott SARS-CoV-2 chemiluminescence microparticle immunoassay (CMIA) that detects anti-nucleocapsid (anti-N) IgG antibodies:

1) A_{neg}: S/C ratios of anti-N IgG for 821 pre-pandemic blood donors in Manaus (negative controls; Figure 3A);
2) $A(0)$: S/C anti-N IgG ratios for 193 infected plasma donors $20 - 50$ days after symptom onset (cases/positive controls; Figure 3A);

3) r: decay rate of $\log_{10}$(S/C) anti-N IgG ratios, defined as the difference in $\log_{10}$(S/C) ratios over the difference in time between longitudinal measurements in 88 convalescent plasma donors (Figure 3B).

4) Θ: The assay's positivity threshold provided by the manufacturer.

In addition to these validation data, Buss *et al.* shared S/C anti-N IgG ratios from 800-900 randomly selected blood donors in Manaus each month between March–October 2020. Note that every month a new subset of blood donors was selected. It is not clear to us, however, whether these subsets are disjoint.

Optimization routine

The log-likelihood function is maximized using the local optimization routine *optim* in the R software (6) constrained to positive real numbers that in sum do not exceed one. The use of a local optimization routine may result in cumulative incidence estimates that only represent a local but not a global maximum of the log-likelihood function. In the simulation studies we use the difference of sequential sensitivity-adjusted seroprevalence estimates as the initial parameter guess and replace potentially arising negative values by 10^{-6} to guarantee positivity. When estimating the cumulative incidence in the real world example from Manaus, we test a large number of initial parameter values, and run the local optimization for a set of initial parameters thereby attempting to find the global maximum. For bootstrapping we use the point estimates as initial parameters.

Codes and data

The method has been implemented in the R-package "kadelka23aje" and is available as a Web Appendix to this article. To install the R package download "kadelka23aje_0.0.0.9000.tar.gz" and install the package in R using the command *install.packages("path_to_file/kadelka23aje_0.0.0.9000.tar.gz", repos = NULL, type = "source")*, where *path_to_file* is replaced with the location of the downloaded file. If an error message about missing packages is received, those packages need to be installed first. Required packages are BB, dplyr, fitdistrplus, magrittr, MASS, mc2d, SimDesign and sn.

Web Appendix 2 – Results

Theoretical results: Sensitivity analysis

The results presented in the article show good agreement between fitted and true cumulative incidences for all 7 test cases. Here, we determine the impact of various assumptions on the performance of the proposed method.

Distribution of recoveries between consecutive surveys

To this point, we assumed uniformly distributed recoveries between any two consecutive studies, i.e. incidence was assumed to be approximately piecewise constant with jumps at the times of the serosurveys. Since incidence is in general not constant, we consider four other options for the distribution of recoveries between surveys. As described in the Web Appendix , we consider (1) all recoveries at the day after the former study, (2) daily recoveries decay exponentially between two studies, (3) daily recoveries grow exponentially between two studies and (4) all recoveries at the day of the latter study. Note, that the same general shape of recovery time distribution is assumed among all time intervals between two surveys, however with varying growth/decay rates between the time intervals under assumptions (2) and (3). Summary statistics of the estimated cumulative incidences from 10000 *in silico* studies together with the true cumulative incidences are shown in Web Figures 2–5 and the method's powers are summarized in Web Table 1 (rows 2–5). As sero-reversion probabilities are estimated based on the assumption that, on average, recovery happens exactly at the midpoint between two surveys, it is not surprising that for test cases 1–5 the method performs best when recoveries are uniformly distributed between any two consecutive surveys. In situations where the majority of recoveries occur early during the time interval between surveys, the method loses power due to under-correction for sero-reversion and hence under-estimation of cumulative incidences while it loses power due to over-correction (over-estimation of cumulative incidences) when recoveries occur late.

Time between surveys

Previously, we assumed that serosurveys are conducted monthly. To investigate how the duration between two surveys influences the performance of the proposed method, the same seven test cases with surveys conducted in the first month and then followed up every 2 months are considered. Precise values of the assumed true per capita new

recoveries between two surveys are summarized in equation (9). While, with powers ranging from 80.4% (test case 4) to 95.2% (test case 2), the method's performance is still good when assuming uniformly distributed recoveries between consecutive surveys, it is reduced compared to when monthly surveys are available (see Web Figure 6 and Web Table 1). However, an increasing delay between consecutive surveys leads to a more pronounced loss of power due to under- or over-correction if infections are not assumed to be uniformly distributed between surveys (compare Web Figures 2–5 with 7–10 and Web Table 1). Lastly, our definition of a successful fit, which allows for a 2% absolute difference between true and estimated cumulative incidence at each time point, results in relatively large statistical powers in all situations for test cases 6 and 7 which are characterized by continuously low incidences.

Correlated antibody peak and decay

There is evidence for a correlation (of varying strength) between peak antibody levels and antibody decay rates after SARS-CoV-2 infections (3; 4). Assuming a uniform distribution of recovery times between any two surveys, we test the method for true underlying Spearman correlations in $\{-0.9, -0.6, -0.3, 0, 0.3, 0.6, 0.9\}$. For the derivation of sero-reversion times from the validation data we assume Spearman correlations in $\{-0.9, -0.6, -0.3, 0, 0.3, 0.6, 0.9\}$ and evaluate the power of the method for each combination of true underlying and assumed Spearman correlation using 300 simulated *in silico* studies. The results in Web Figure 11 show that in general the method performs well if assumed and true correlation are similar. In situations where a large fraction of cases occur early during the study period (test case 1, 4 and 5) the method fails to estimate cumulative incidence reliably when assuming a correlation that varies significantly from the true underlying correlation. This is due to inadequate probabilities of sero-reversion which lead to large over- or under-correction for sero-reversion. By contrast, the impact of a discrepancy between true underlying and assumed correlation is lower with powers above 40% for all combinations of true and assumed correlations in situations where the majority of cases occur late in the study period (test cases 2 and 3) or when incidence is low throughout the entire study period (test cases 6 and 7).

Number of individuals per survey

An important factor influencing how well the method estimates the cumulative incidence is the sample size. Web Figure 12 shows, for all test cases, the power determined from 3000 simulated surveys versus the sample size, where the sample size is defined as the fixed number of participants in each survey within a study. For sample sizes between 10 and $3162 \approx 10^{3.5}$ individuals per survey the power increases from below 15% to 92-95% for all test cases. Including more than 3162 individuals per survey does not further improve the power in any of the seven test cases. How fast the power grows with increasing sample sizes depends on the test case. While 100 participants per survey are enough to reach close to 50% power in test cases 1, 2, 5 and 6, the power for test cases 3, 4 and 7 is below 25%. Note that the size of the control data sets were kept constant and only the number of participants per survey in the *in silico* studies was varied. Furthermore, we assumed a uniform distribution of recovery times between any two surveys and disjoint sets of participants at the surveys within one study. If instead, a constant cohort is followed through time, the antibody levels of infected individuals are pre-determined for future surveys and hence the effective sample size of measurements that yield new information shrinks with each survey. This results in reduced powers compared to when using disjoint survey populations of the same size (see Web Figure 13. However, for large sample sizes the same powers approach the same limit.

Varying probabilities of sero-reversion

We compare the performance of the proposed method when using empirically derived probabilities of sero-reversion with the following alternatives: (1) no sero-reversion, (2) probabilities of sero-reversion obtained under the assumption that times from sero-conversion to sero-reversion are exponentially, (3) Weibull- or (4) uniformly distributed. For all distributions, we assume that the median of the respective distribution equals the empirically-derived median of sero-reversion times, Φ_{emp}, (in days).

(1) No sero-reversion. In this case all sero-reversion probabilities are set to zero, i.e. $p_{rev}^{|pos.con}(t) = 0$ for all t.

(2) Exponentially-distributed sero-reversion times. Let $X \sim Exp(\lambda)$ be a random variable describing sero-reversion times (in days past seroconversion). Let $f_X(x; \lambda)$ be the corresponding density function. Since median$[X] = \ln(2)/\lambda$ and median$[X] = \Phi_{emp}$, we find $\lambda = \ln(2)/\Phi_{emp}$. The probability to sero-revert by time x is given by $p_{exp}(x) = F_X(x; \lambda = \ln(2)/\Phi_{emp})$. Furthermore, this distribution of sero-reversion times results in an exponential decay of the probabilities to sero-revert within a given month past seroconversion, which corresponds to Buss *et al.*'s assumption. ($\int_{30n}^{30(n+1)} \lambda e^{-\lambda x} dx = ce^{-30\lambda n}$, where $c = (1 - e^{-\lambda})$ and $n = 0, \dots, M - 1$)

(3) Weibull-distributed sero-reversion times. Let $X \sim Weibull(\lambda, k)$ be a random variable describing sero-reversion times (in days), with corresponding probability density function $g_X(x; \lambda, k)$. Then the probability to sero-revert by time x is given by $p_{Weibull}(x) = G_X(x; \lambda, k)$. Here, λ is the scale and k the shape parameter of the

Weibull-distibution. Using

$$\text{median}[X] = \lambda(\ln(2))^{1/k},$$
$$\text{stdev}[X] = \lambda\sqrt{\Gamma\left(1+\frac{2}{k}\right) - \left(\Gamma\left(1+\frac{2}{k}\right)\right)^2}$$

we find

$$\text{stdev}[X] = \frac{\text{median}[X]}{\ln(2)^{1/k}}\sqrt{\Gamma\left(1+\frac{2}{k}\right) - \left(\Gamma\left(1+\frac{2}{k}\right)\right)^2}. \tag{10}$$

For given median$[X]$ and stdev$[X]$, the shape parameter k can be found by solving equation (10) for $k \in (0, \infty)$. The scale parameter is then given by $\lambda = \text{median}[X]/(\ln(2))^{1/k}$. We consider two different assumptions: (3a) The standard-deviation of the Weibull-distribution is fixed at 50 days, as it was assumed by Shoida *et al.*. (3b) The standard-deviation of the Weibull-distribution is fixed at 150 days. In both cases we assume median$[X] = \Phi_{emp}$. The sero-reversion probabilities will be referred to as $p_{Weilbull}$ in case (3a) and $p_{Weibull150}$ in case (3b) is assumed. **(4) Uniformly-distributed sero-reversion times.** Let $X \sim Unif(0, max)$ be a random variable describing sero-reversion times (in days past seroconversion). Let $h_X(x; max)$ be the corresponding density function. Median$[X] = \Phi_{emp}$ implies $max = 2\text{median}[X] = 2\Phi_{emp}$. Then the probability to sero-revert by time x is given by $p_{unif}(x) = H_X(x; max = 2\Phi_{emp})$.

A certain fraction of seroconverters never sero-reverts. The empirically-derived sero-reversion times have a point mass at infinity. If we assume $\lim_{t\to\infty} p_{rev}^{|pos,con}(t) = p \in (0, 1)$ then $p_{rev}^{|pos,con}(\infty) = 1 - p$. We adjust the probablities of sero-reversion that were derived assuming exponentially, Weibull- and uniformly distributed sero-reversion times accordingly: $\tilde{p}_j(x) = p \times p_j(x)$, where $j \in \{exp,\ Weibull,\ Weibull150,\ unif\}$.

Web Figure 14 (left panel) shows the median of the empirically-derived sero-reversion probabilities $p_{emp}(t) = p_{rev}^{|pos,con}(t)$, $t = 0.5, \ldots, 16.5$ months, from 3000 validation data sets together with the medians of $\tilde{p}_{exp}(t)$, $\tilde{p}_{Weibull}(t)$, $\tilde{p}_{Weibull150}(t)$ and $\tilde{p}_{unif}(t)$. The right-hand panel shows the corresponding probabilities to sero-revert within a given time-interval past sero-conversion. When assuming that sero-reversion times are uniformly distributed with median Φ_{emp} or Weibull-distributed with standard deviation 150days and median Φ_{emp} the probabilities of sero-reversion are very similar to the empirically-derived ones for the first 5.5 months. By contrast, if sero-reversion times are assumed to be exponentially distributed with median Φ_{emp} or Weibull-distributed with standard deviation of 50 days and median Φ_{emp}, sero-reversion probabilities are above or below the empirically derived ones for the first 5.5 months, respectively. For $t > 5.5$ months $\tilde{p}_{exp}(t)$ is closest to $p_{emp}(t)$, while all other assumptions (Weibull-distribution with standard deviation 50 or 150 days or uniform distribution) result in significantly larger probabilities of sero-reversion.

Variations in sero-reversion probabilities result in variations of estimated cumulative incidences. In scenarios where only the first 9 months of an epidemic are considered (test cases 1,2,3,4,6), the method performs almost equally well when using $\tilde{p}_{Weibull,150}$ or $\tilde{p}_{unif}$ compared to p_{emp} (see Web Figure 15 and Web Table 2), while cumulative incidences are underestimated (due to under-correction for sero-reversion) when assuming $\tilde{p}_{Weibull,50}$ or no sero-reversion and overestimated when assuming $\tilde{p}_{exp}$. Despite the general shift towards a small overestimation when assuming $\tilde{p}_{exp}$, the method often still succeeds at estimating cumulative incidences (see powers in Web Table 2). When observing the start of an epidemic for a longer time, such as in test case 5 (15months), assuming $\tilde{p}_{exp}$ becomes the most powerful alternative to the empirically-derived sero-reversion times, since for large t, $\tilde{p}_{exp}(t)$ is the most similar to $p_{emp}(t)$. On the other hand, assuming uniformly distributed sero-reversion times, which yields good cumulative incidence estimates when only the initial 9 months are considered, results in a very low power for longer observation periods due to overestimation of and over-correction for sero-reversions at later times.

Real world example

Correlation between peak and decay rate of antibody level

In contrast to what has been observed in (3) and (4), the longitudinal data from the 81 convalescent plasma donors in Buss et al.'s validation data set presents some evidence for a negative correlation of the peak antibody level and antibody decay rate ($A(0)$ and r in equation (1), Spearman $R = -0.31$, $P = 0.005$). This suggests a slower decay of antibody titers in individuals with initially high titer, a faster decay in those starting at low levels, and hence a wider distribution of sero-reversion times compared to the uncorrelated case (not shown). For demonstration purposes, we consider the effects of a wide range of correlation coefficients on the profile of the probability to sero-revert in the first $0 \leq a \leq 9$ months past recovery (see Web Figure 18A). Varying the distribution of sero-reversion times, and consequently the probabilities of sero-reversion by assuming a correlation between peak anti-N S/C ratio and its exponential decay rate does not significantly alter any cumulative incidence estimate (see Web Figure 18B). This indicates that our results are robust to possible correlations between peak level and decay rate of antibodies.

Different control data

The positive control data used in the main analysis to derive the sero-reversion probability stems from convalescent patients and is only representative of symptomatic, non-hospitalised COVID-19 cases. Antibody levels, however, vary with disease severity (7; 8) and blood donors in Manaus have had mostly mild or asymptomatic infections. Thus, using repeated blood donors that have been infected as positive controls can reduce the mismatch between control data used for the derivation of sero-reversion times and survey data. However, the history of infection (in terms of the gold standard of a positive PCR test) is in general not known for blood donors. Here, using data provided by (9), we derived the sero-reversion probablities from repeated blood donors with a positive antibody test in Manaus. The distribution of peak antibody levels is fitted to the maximal values of measured antibody levels of 432 manauense blood donors who (1) had a positive antibody test at two or more donation time points and (2) whose maximal antibody level was measured in May 2020. In this context, as in (9), a positive antibody test is defined as a S/C value above 0.49. Restriction to donors with peak measurement in May 2020 guarantees that antibody levels have not decayed for very long since the epidemic only set off in March 2020. Individuals whose peak measurement occurred before May 2020 were ignored since the measured antibody levels were on average far below those measured for individuals with peak measurements in May, which may be a hint that these individuals had not reached their peaks (not shown). However, restriction to repeat donors with multiple positive tests may bias towards those with higher peak measurements and neglect those with below threshold peak antibody levels or close to cutoff antibody levels, whose antibody levels, even in a short time, may have dropped below the threshold and hence have no second positive test. Furthermore, uninfected repeat blood donors with above threshold antibody levels (false positives) may accidentally be included. Lastly, it is important to note that any measured antibody level is below the actual peak of the respective individual (except for possible measurement errors), and thus fitting the distribution of peak levels to the peak measured antibody levels biases towards too low values. Assuming exponential decay of antibody levels after their peak, the distribution of anti-N IgG decay rates was derived from 2112 repeat blood donors in Manaus with peak antibody level above the threshold for sero-positivity and at least one measurement after that peak. For each such donor, the base-10 decay rate d between the measured peak antibody level, A_{peak}, and the first antibody level after sero-reversion, or if sero-reversion is not observed, the last measured antibody level, A_{end}, is defined as

$$d = \frac{\log_{10}(A_{peak}) - log_{10}(A_{end})}{t_{peak} - t_{end}}.$$

Web Figure 16 compares the obtained cumulative distributions of sero-reversion times and fitted cumulative incidences in Manaus with those obtained when using convalescent patients as the positve control group (blue vs red curves). We observe that sero-reversion occurs earlier in the repeated blood donors, which results in increased cumulative incidence at most times. In October 2020, the estimated cumulative incidence is 54.6% (95% confidence region 50-59.6%), and thus still significantly below Buss *et al.*'s estimate of 76%.

References

[1] De Graaf W, Kretzschmar M, Teunis P, et al. A two-phase within-host model for immune response and its application to serological profiles of pertussis. Epidemics, 9:1–7, 2014.

[2] Hamady A, Lee J, and Loboda ZA. Waning antibody responses in COVID-19: what can we learn from the analysis of other coronaviruses? Infection, pages 1–15, 2021.

[3] Steenhuis M, van Mierlo G, Derksen NI, et al. Dynamics of antibodies to SARS-CoV-2 in convalescent plasma donors. Clin Transl Immunology, 10(5):e1285, 2021.

[4] Xia W, Li M, Wang Y, et al. Longitudinal analysis of antibody decay in convalescent COVID-19 patients. Sci Rep, 11(1):1–9, 2021.

[5] Buss LF, Prete CA, Abrahim CM, et al. Three-quarters attack rate of SARS-CoV-2 in the Brazilian Amazon during a largely unmitigated epidemic. Science, 371(6526):288–292, 2021.

[6] R Core Team . R: A Language and Environment for Statistical Computing. R Foundation for Statistical Computing, Vienna, Austria, 2020.

[7] Hashem AM, Algaissi A, Almahboub SA, et al. Early humoral response correlates with disease severity and outcomes in COVID-19 patients. Viruses, 12(12):1390, 2020.

[8] Van Elslande J, Oyaert M, Lorent N, et al. Lower persistence of anti-nucleocapsid compared to anti-spike antibodies up to one year after SARS-CoV-2 infection. Diagn Microbiol Infect Dis, 103(1):115659, 2022.

[9] Prete Jr CA, Buss LF, Whittaker C, et al. SARS-CoV-2 antibody dynamics in blood donors and COVID-19 epidemiology in eight Brazilian state capitals: A serial cross-sectional study. Elife, 11:e78233, 2022.

Web Tables

Web Table 1: Powers (in %) of the method for all test cases, one months (1M) or two months (2M), and all five possibilities for the temporal distributions of recoveries between any two surveys

Delay Surveys	Distribution Between Surveys	Test Case 1	Test Case 2	Test Case 3	Test Case 4	Test Case 5	Test Case 6	Test Case 7
1M	uniform	93.20	96.10	90.60	87.40	89.80	92.80	90.00
1M	early (1)	68.80	78.10	78.60	68.00	65.50	94.10	91.70
1M	decreasing (2)	92.10	95.10	90.10	85.70	88.90	93.50	91.40
1M	increasing (3)	90.00	94.00	87.60	83.10	84.50	92.20	88.80
1M	late (4)	65.90	79.90	75.10	62.20	61.60	90.90	84.20
2M	uniform	90.10	95.20	83.40	80.80	88.30	94.00	86.20
2M	early (1)	20.70	33.00	39.30	23.80	21.90	94.30	78.60
2M	decreasing (2)	67.50	79.90	71.70	62.70	67.80	94.70	83.80
2M	increasing (3)	61.60	79.80	67.60	58.40	60.90	92.60	81.50
2M	late (4)	20.00	46.70	39.90	24.50	20.80	90.20	73.10

Web Table 2: Powers obtained from fitting 3000 *in silico* studies using the proposed method with four different probabilities of sero-reversion.

Test scenario	1	2	3	4	5	6	7
empirical	94.60	96.40	92.30	89.20	90.00	94.00	90.60
zero reversion	0.00	0.00	0.00	0.00	0.00	85.30	32.20
exponential	82.50	82.00	83.40	74.90	87.60	93.00	89.20
Weibull	2.90	21.00	54.10	22.00	0.30	93.80	82.00
Weibull150	92.50	95.70	90.30	86.10	83.20	93.90	88.10
uniform	91.20	95.30	90.60	85.70	25.90	93.40	76.50

Web Figures

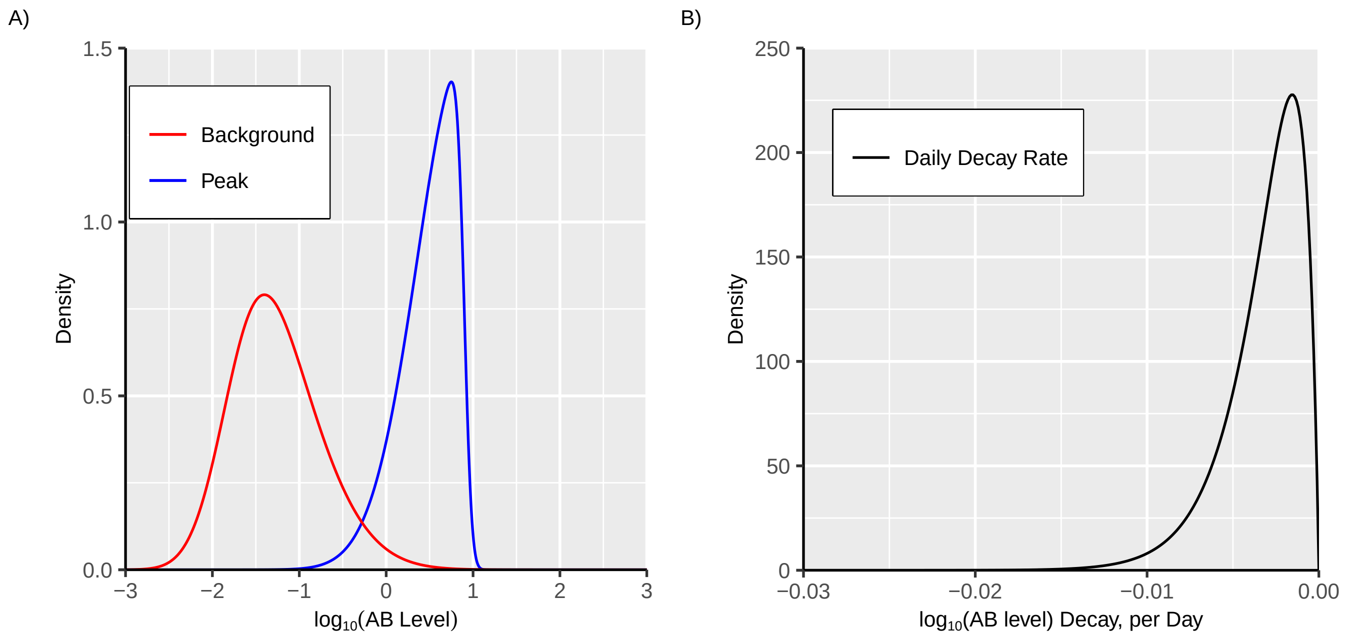

Web Figure 1: Assumed true distributions of A) peak and background antibody levels and B) antibody decay rates.

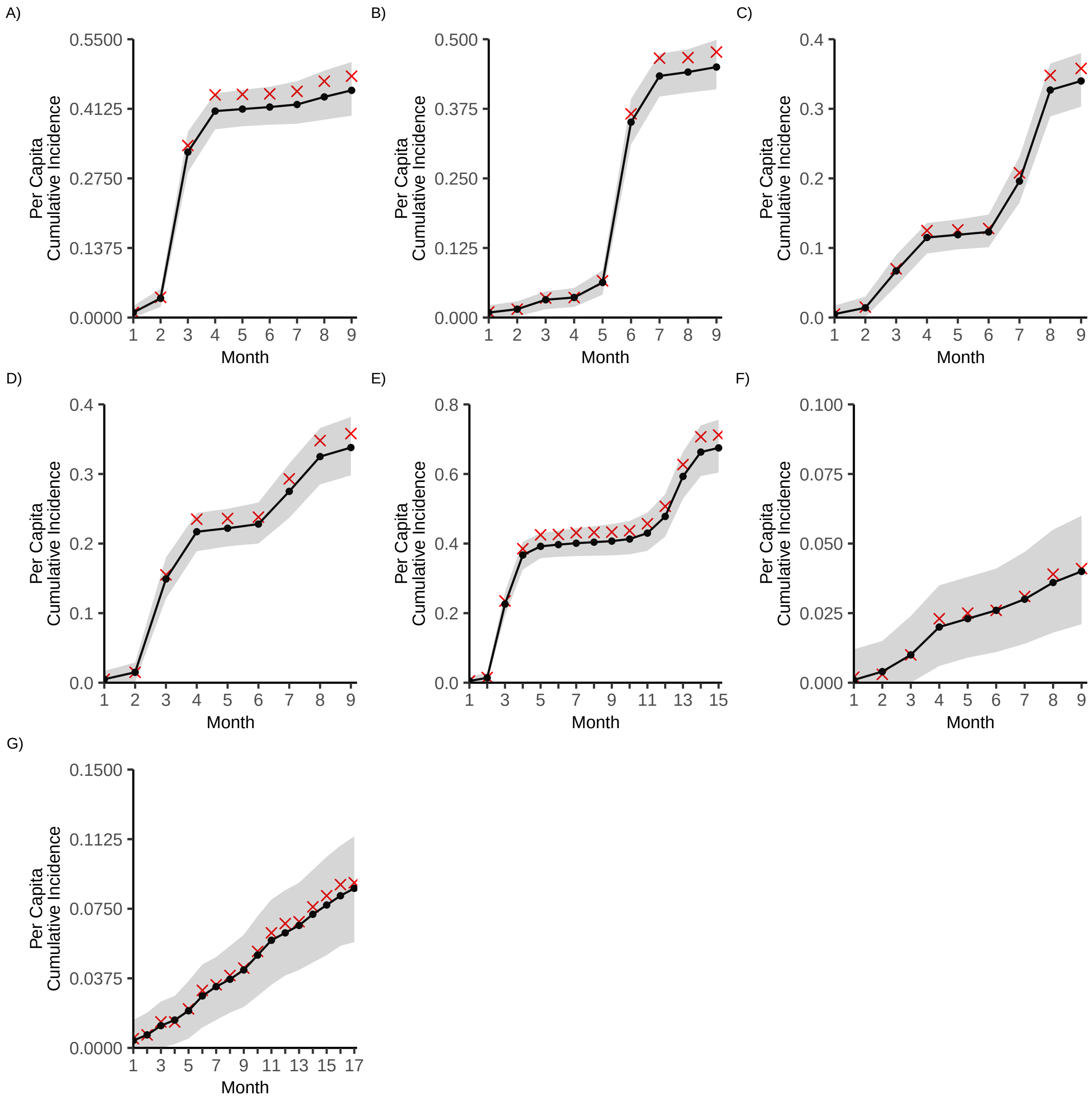

Web Figure 2: True cumulative incidences (red) and median of fitted cumulative incidences from $N_{sim} = 10000$ *in silico* studies (black) with all recoveries at the day past the previous survey and a delay of one month between surveys for test cases 1-7 (A)-G)). The shaded gray region is bounded by the 2.5% and 97.5% quantiles of the estimated cumulative incidences.

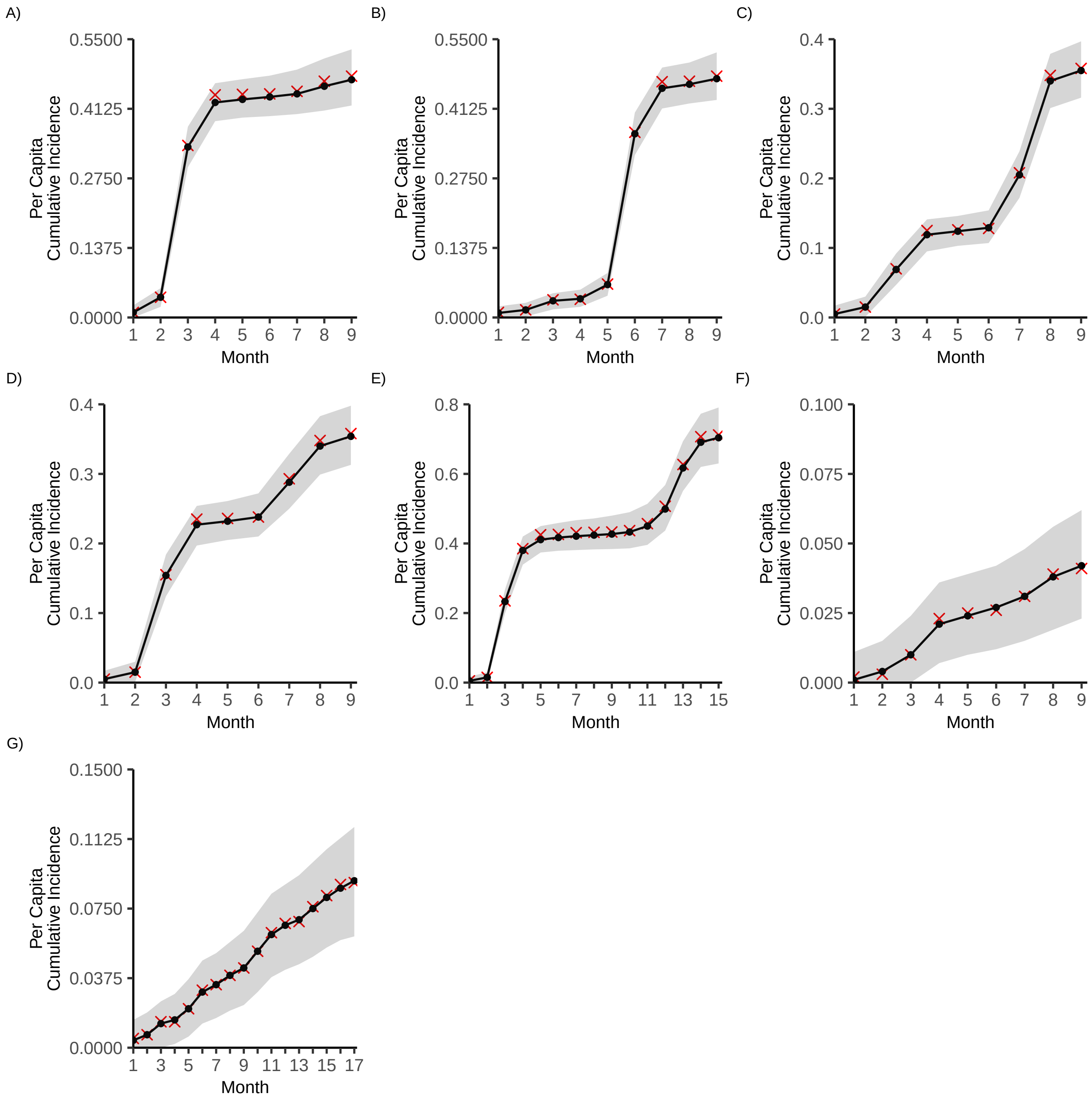

Web Figure 3: True cumulative incidences (red) and median of fitted cumulative incidences from $N_{sim} = 10000$ *in silico* studies (black) with exponentially decreasing numbers of daily recoveries on the intervals between surveys and a delay of one month between surveys. The shaded gray region is bounded by the 2.5% and 97.5% quantiles of the estimated cumulative incidences.

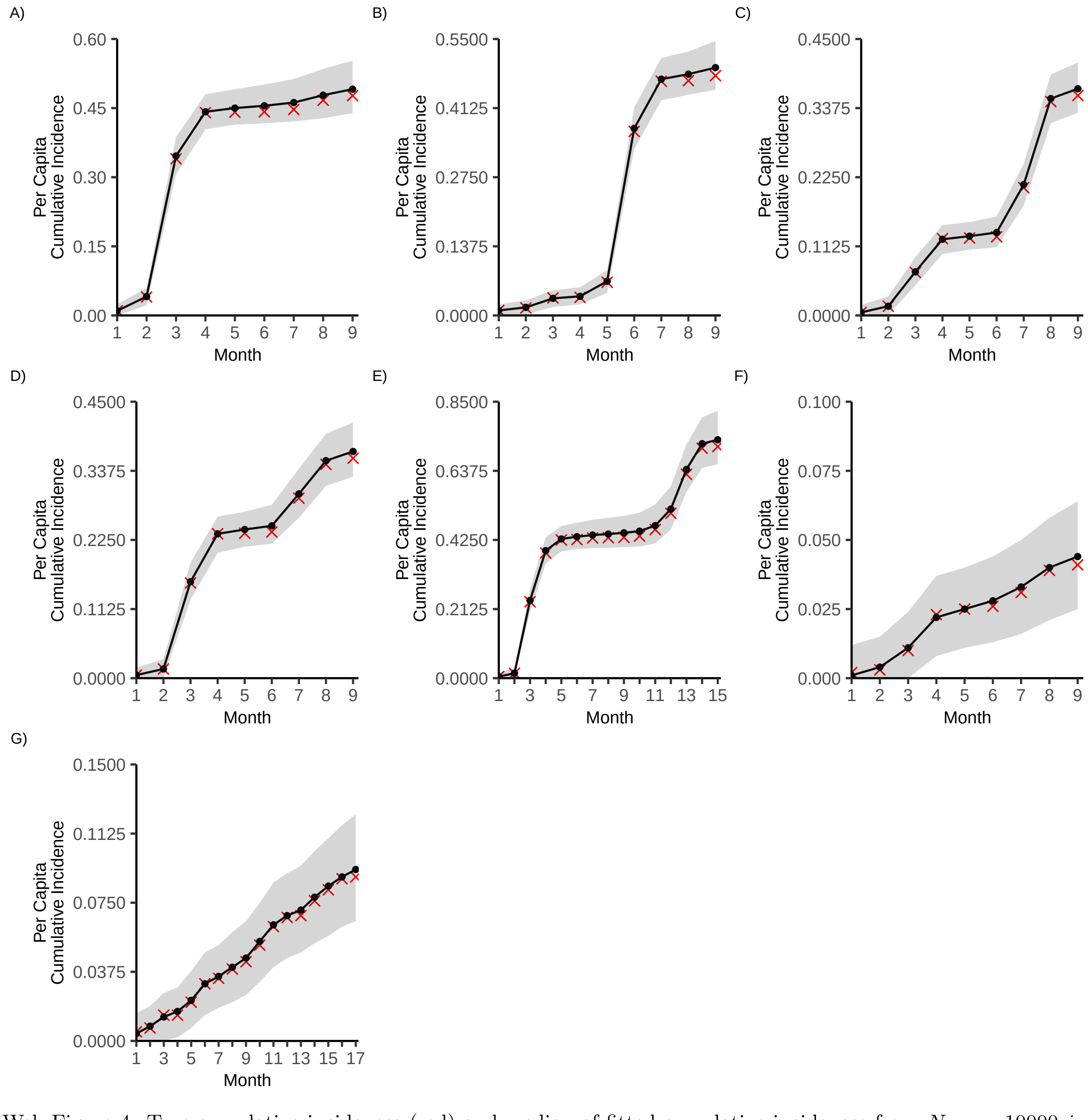

Web Figure 4: True cumulative incidences (red) and median of fitted cumulative incidences from $N_{sim} = 10000$ *in silico* studies (black) with exponentially increasing numbers of daily recoveries on the intervals between surveys and a delay of one month between surveys. The shaded gray region is bounded by the 2.5% and 97.5% quantiles of the estimated cumulative incidences.

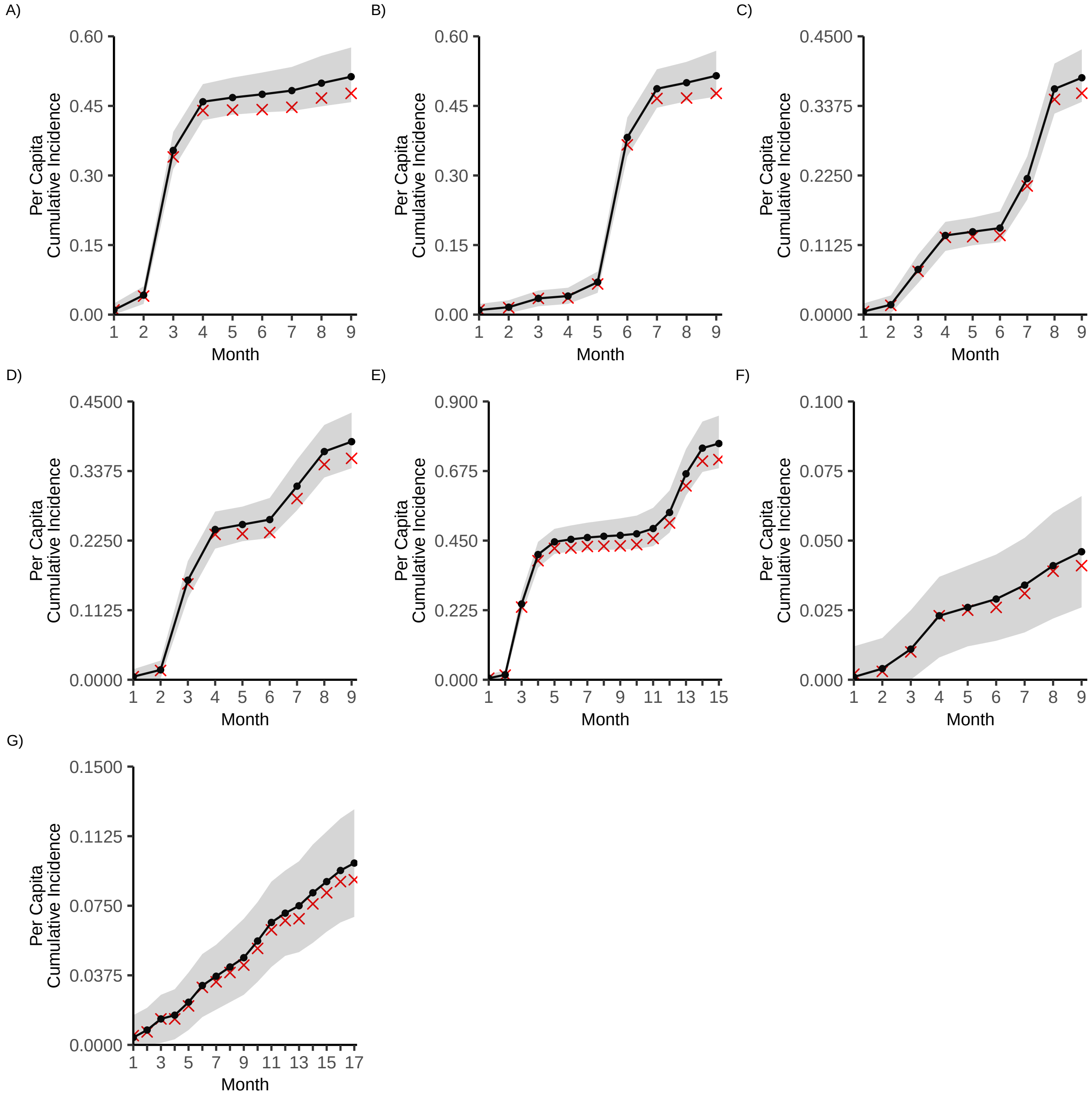

Web Figure 5: True cumulative incidences (red) and median of fitted cumulative incidences from $N_{sim} = 10000$ *in silico* studies (black) with all recoveries at the days of the surveys and a delay of one month between surveys. The shaded gray region is bounded by the 2.5% and 97.5% quantiles of the estimated cumulative incidences.

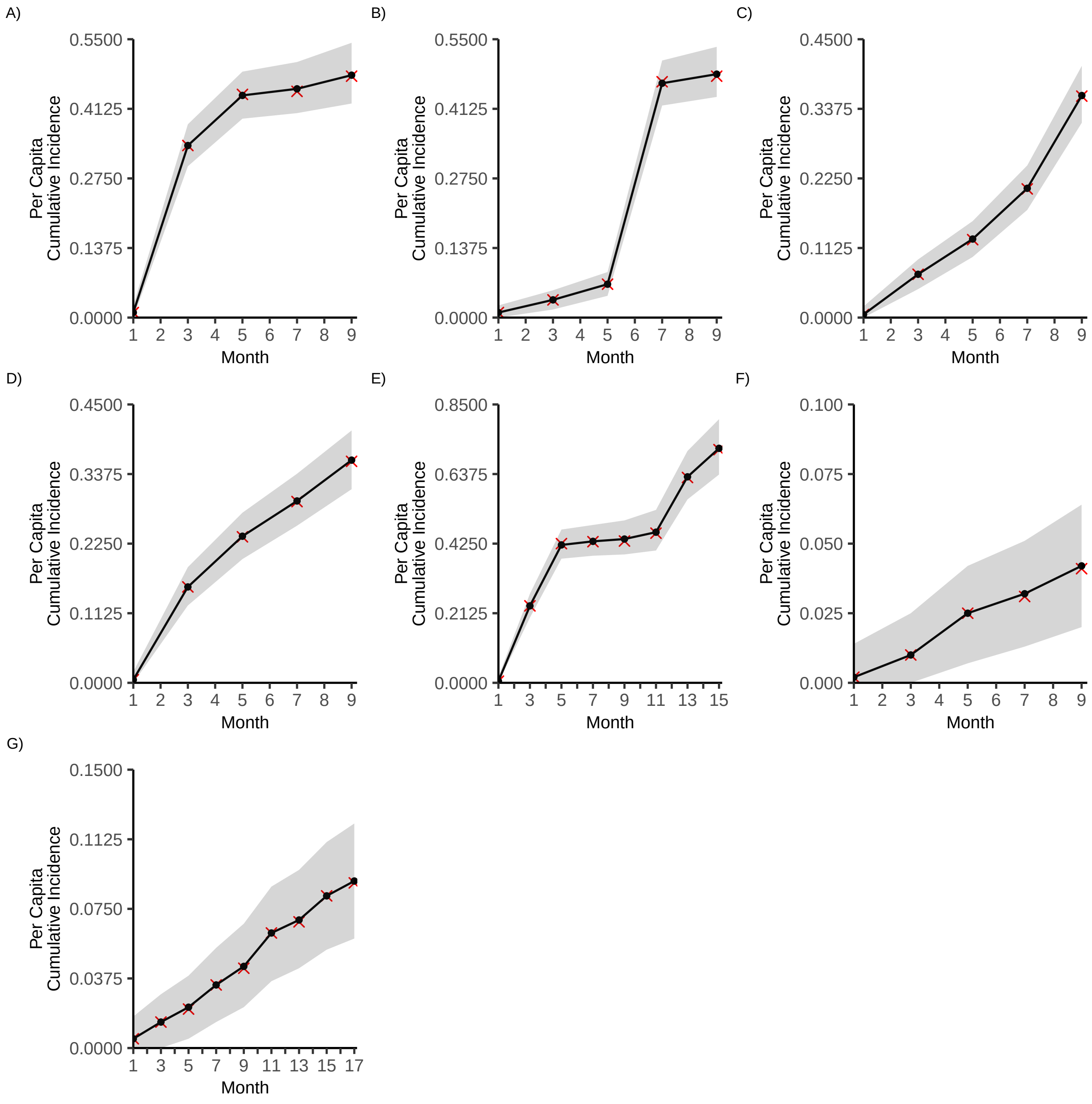

Web Figure 6: True cumulative incidences (red) and median of fitted cumulative incidences from $N_{sim} = 10000$ *in silico* studies (black) with uniformly distributed recovery times between sequential surveys and a delay of two month between surveys. The shaded gray region is bounded by the 2.5% and 97.5% quantiles of the estimated cumulative incidences.

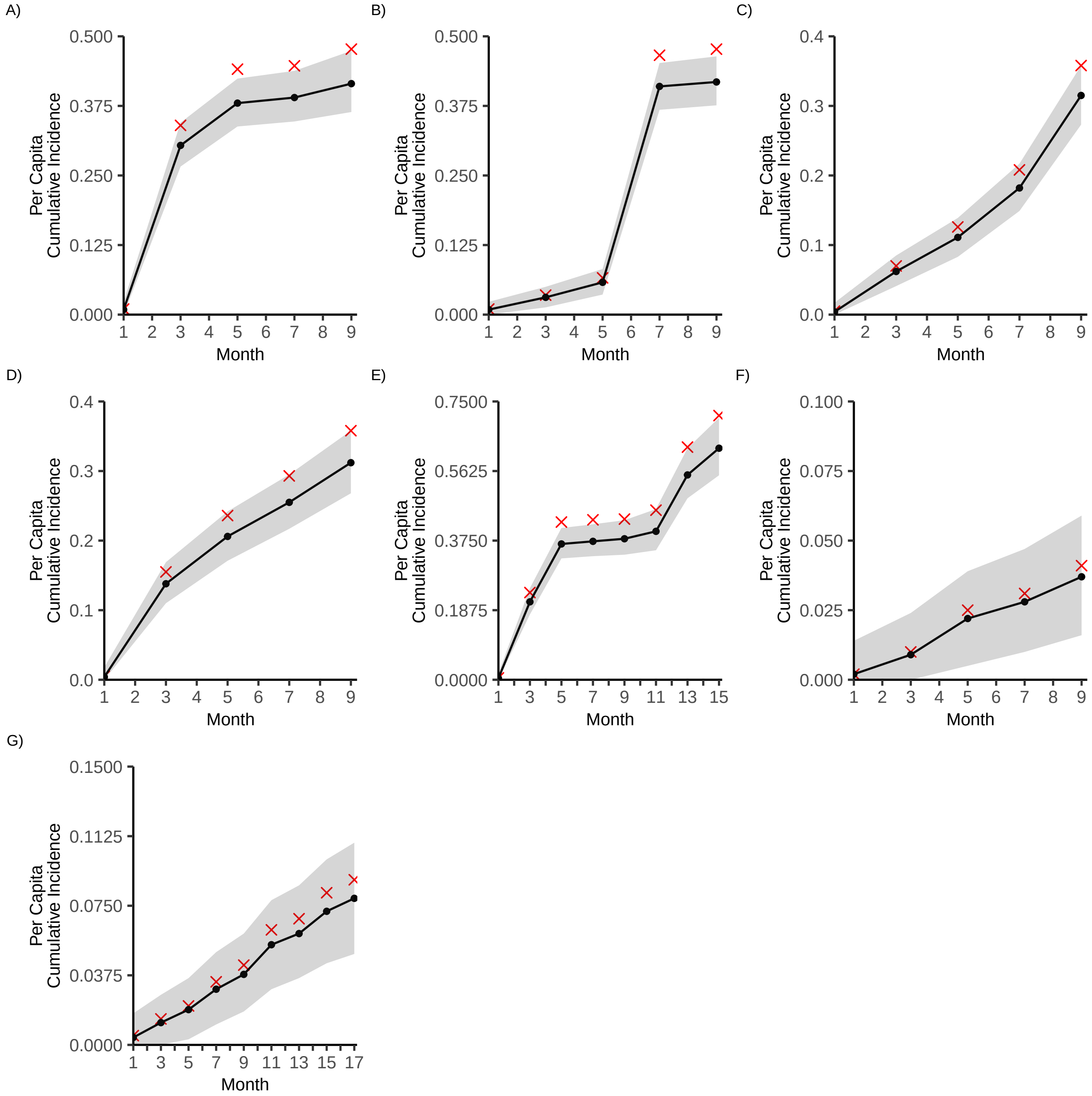

Web Figure 7: True cumulative incidences (red) and median of fitted cumulative incidences from $N_{sim} = 10000$ *in silico* studies (black) with all recoveries at the day past the previous survey and a delay of two month between surveys. The shaded gray region is bounded by the 2.5% and 97.5% quantiles of the estimated cumulative incidences.

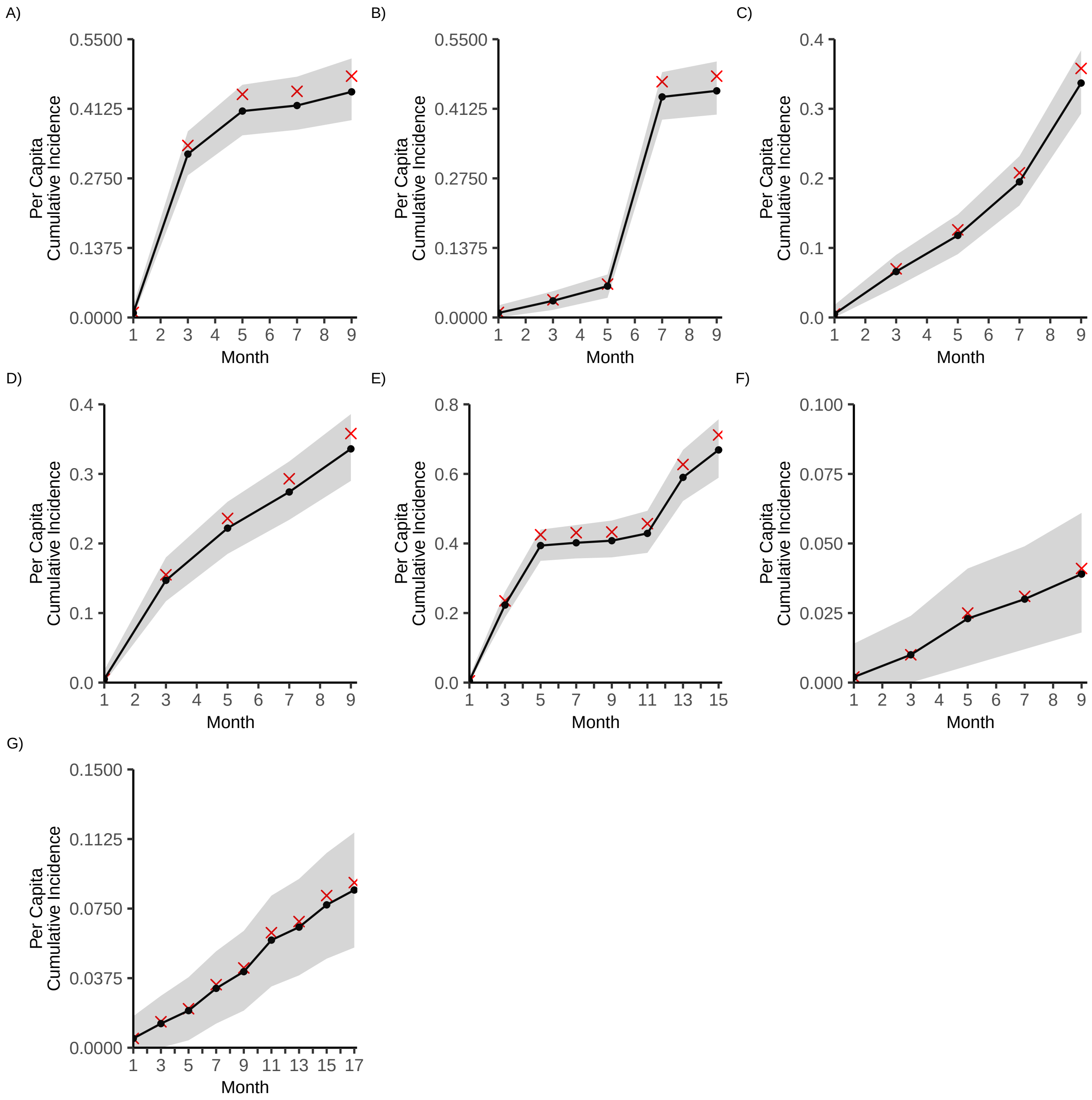

Web Figure 8: True cumulative incidences (red) and median of fitted cumulative incidences from $N_{sim} = 10000$ *in silico* studies (black) with exponentially decreasing numbers of daily recoveries on the intervals between surveys and a delay of two month between surveys. The shaded gray region is bounded by the 2.5% and 97.5% quantiles of the estimated cumulative incidences.

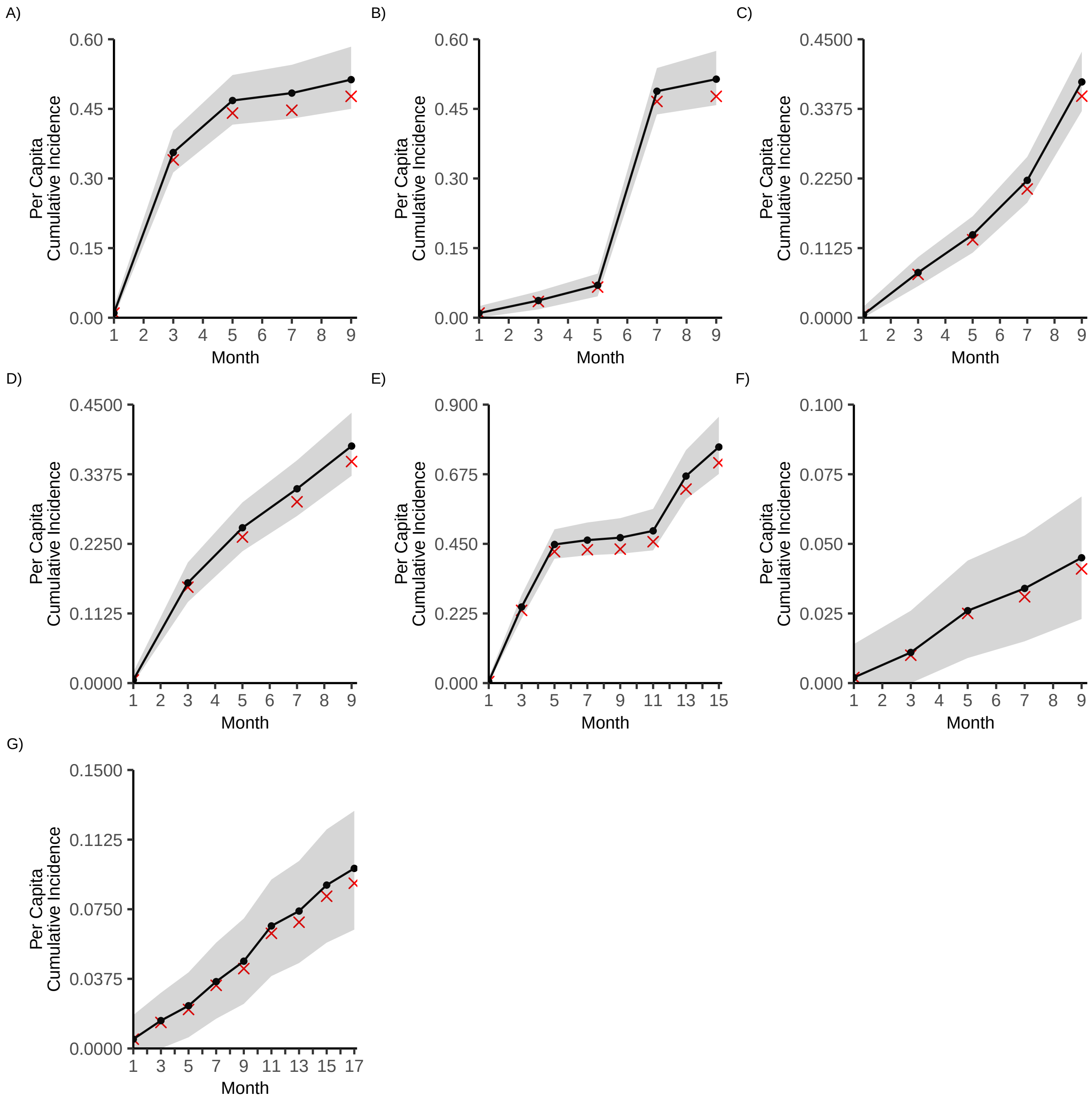

Web Figure 9: True cumulative incidences (red) and median of fitted cumulative incidences from $N_{sim} = 10000$ *in silico* studies (black) with exponentially increasing numbers of daily recoveries on the intervals between surveys and a delay of two month between surveys. The shaded gray region is bounded by the 2.5% and 97.5% quantiles of the estimated cumulative incidences.

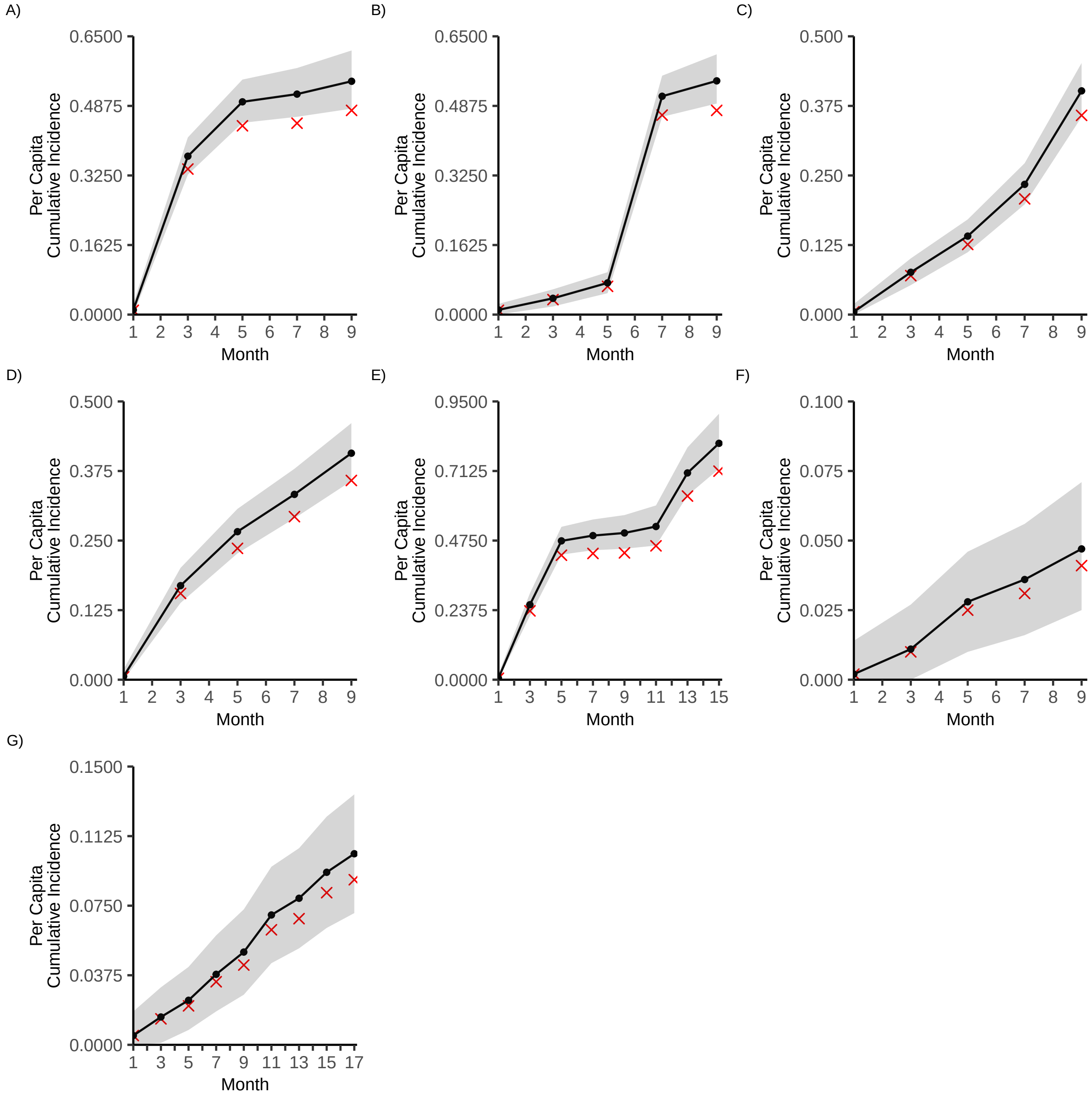

Web Figure 10: True cumulative incidences (red) and median of fitted cumulative incidences from $N_{sim} = 10000$ *in silico* studies (black) with all recoveries at the days of the surveys and a delay of two month between surveys. The shaded gray region is bounded by the 2.5% and 97.5% quantiles of the estimated cumulative incidences.

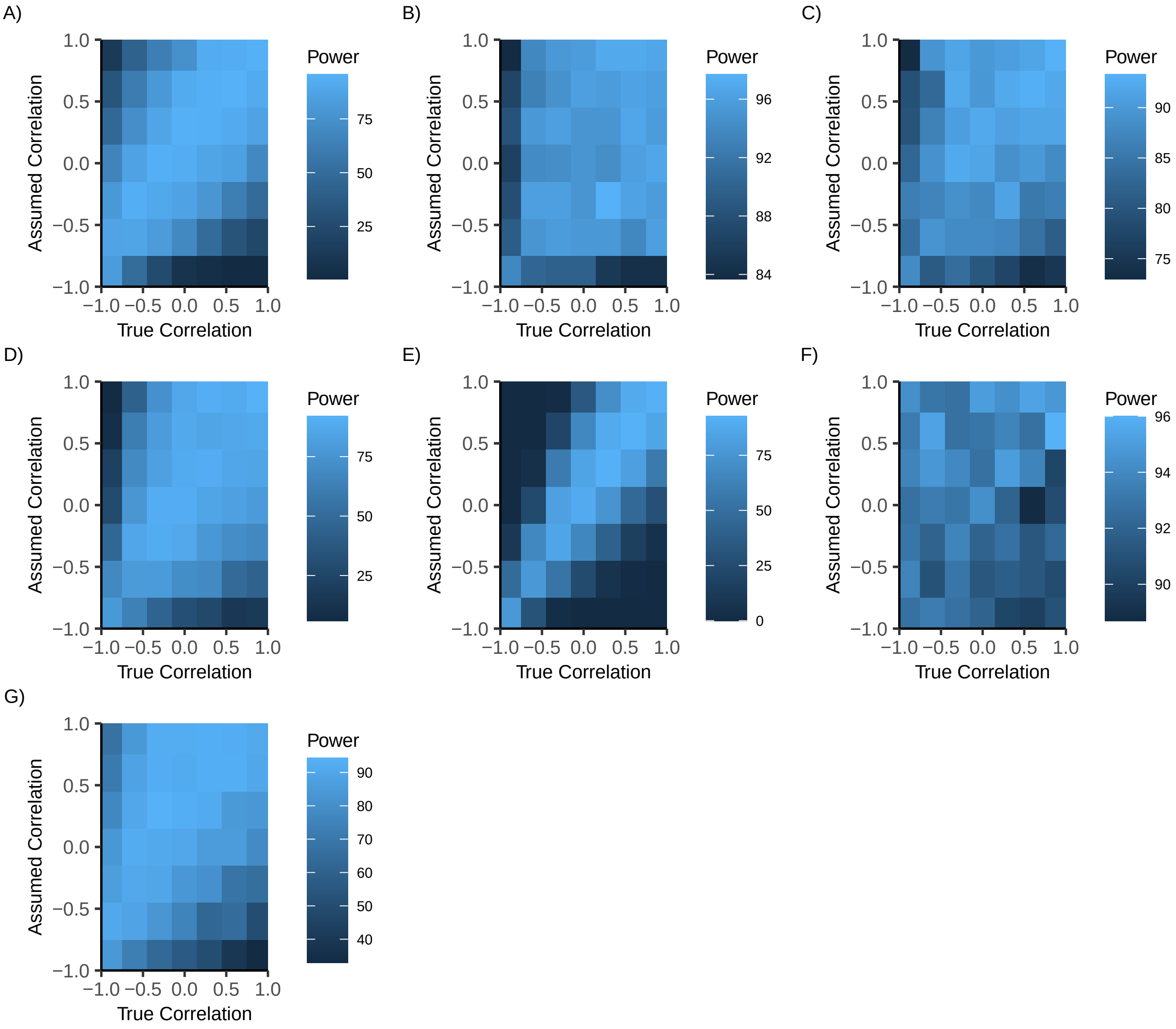

Web Figure 11: Powers for various combinations for true and assumed correlations between antibody peaks and decay rates in SARS-CoV-2-infected individuals. The size of each survey within each of the 300 simulated studies ranged between 800 and 900 individuals.

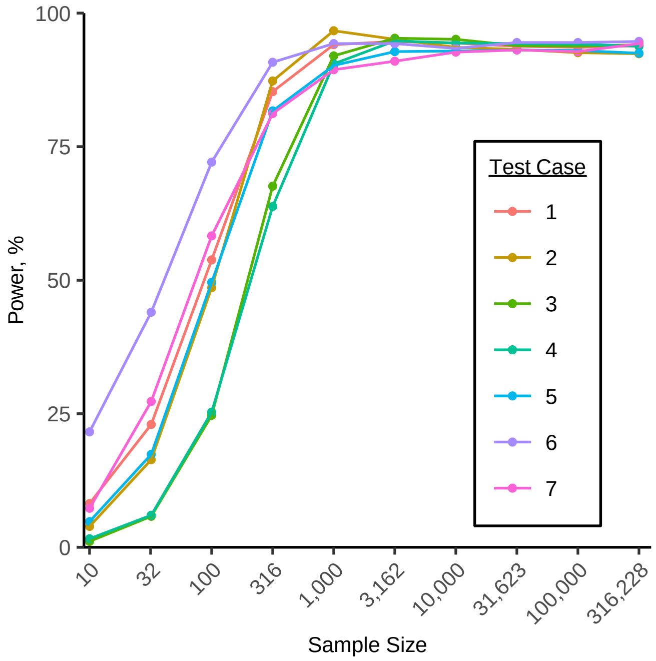

Web Figure 12: Power versus number of individuals surveyed at each time point. Power was determined using 3000 simulated studies.

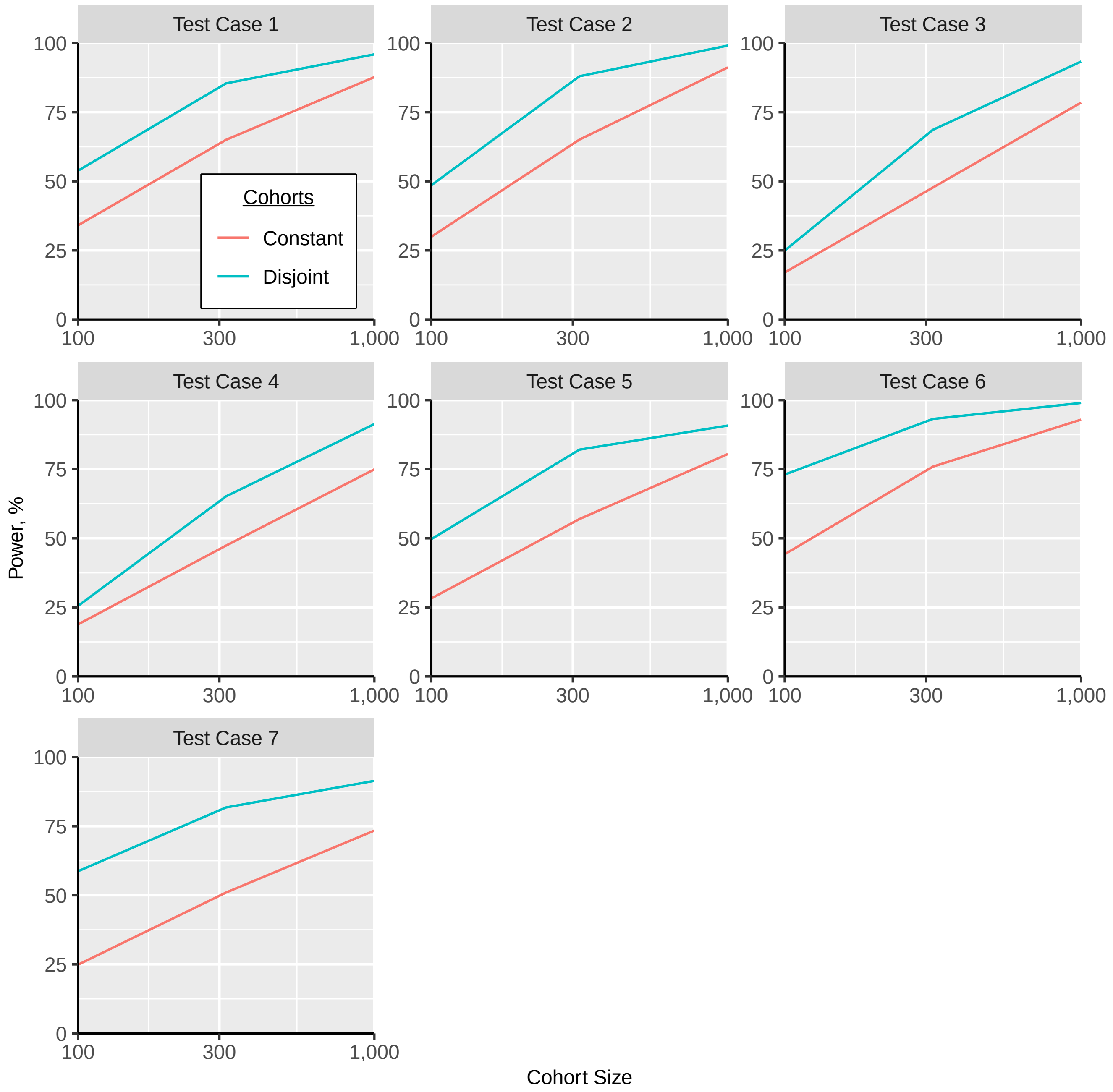

Web Figure 13: Power of proposed method for the various test cases and sample sizes per survey (3000 in silico studies simulated for each test case and each sample size) when assuming a disjoint cohorts (blue) or on constant cohort (red) tested at each sero-survey within one in silico study.

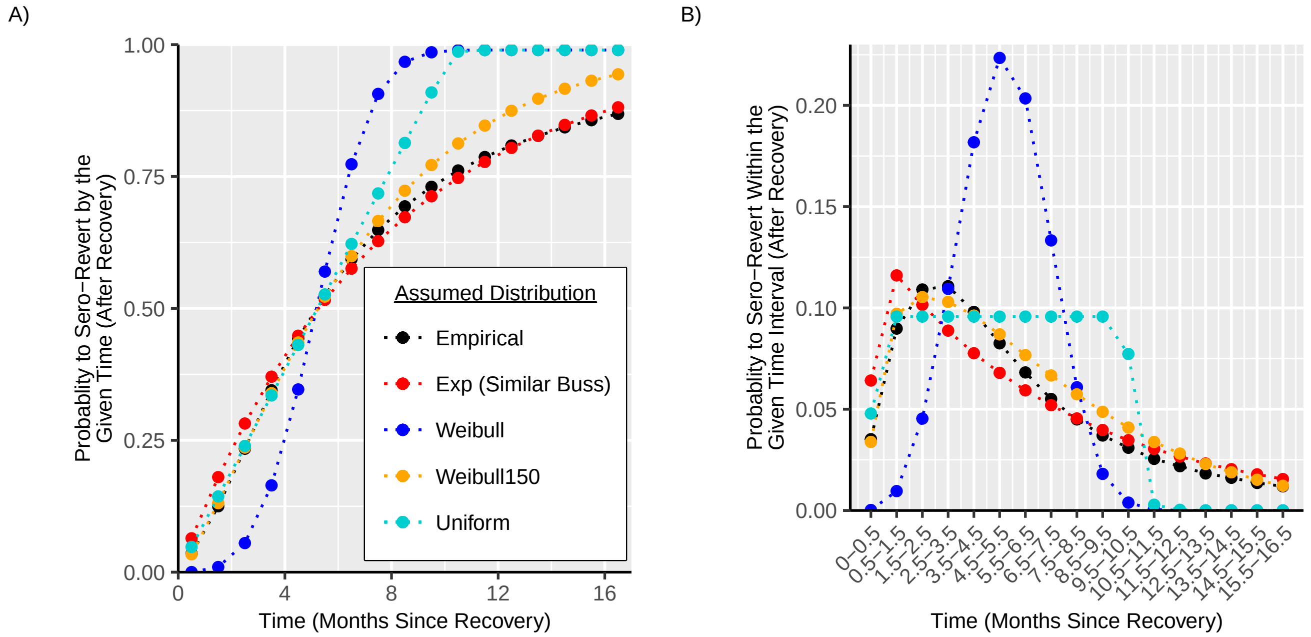

Web Figure 14: Median of probabilities to sero-revert by the given time (left panel) or within the given time interval (right panel) derived from 3000 validation data sets under various assumptions for the first 16.5 months.

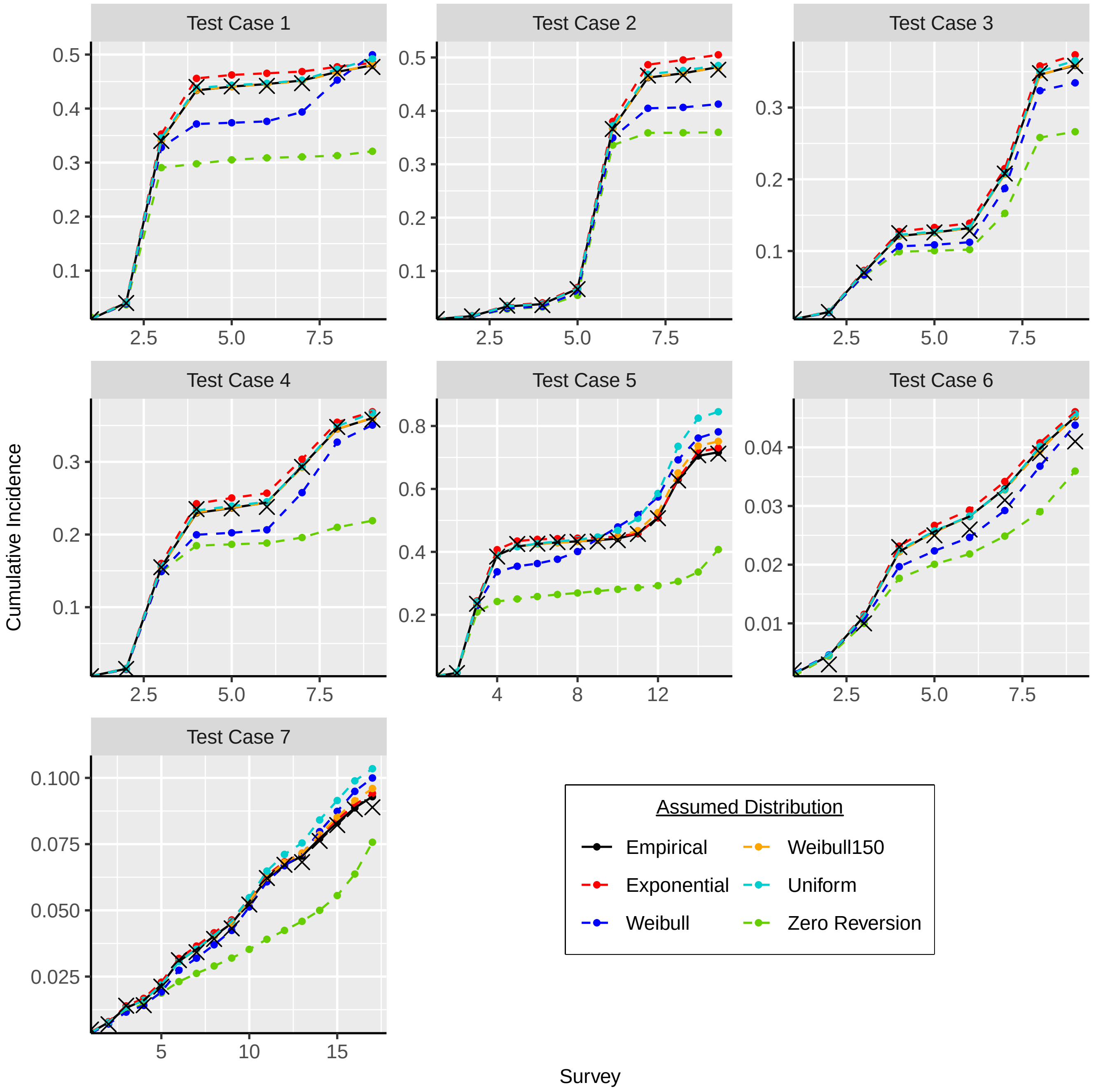

Web Figure 15: Median fitted cumulative incidences from 3000 *in silico* studies per test case under various assumptions on sero-reversion probabilities. Crosses mark the true cumulative incidences.

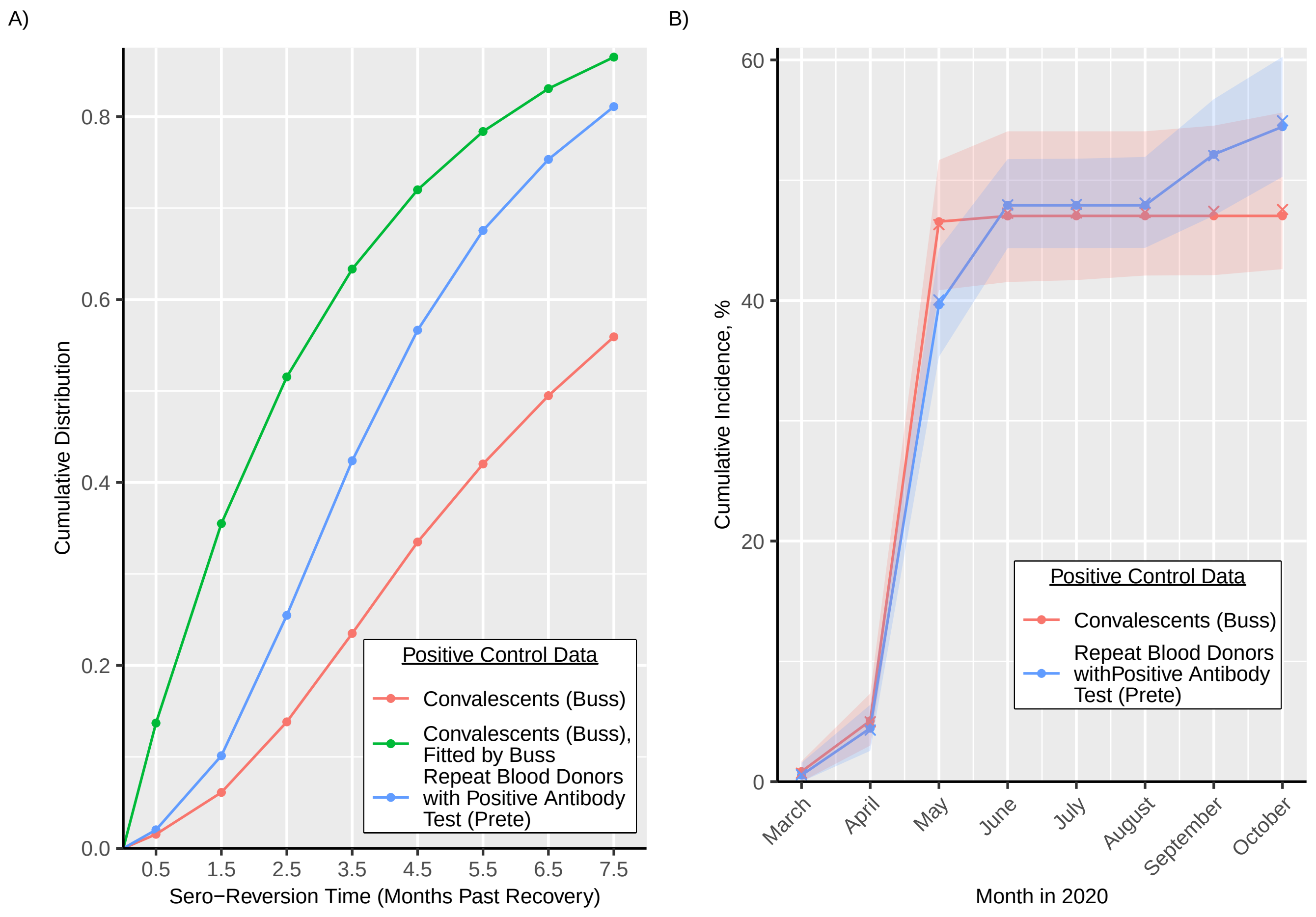

Web Figure 16: A) Cumulative distribution of sero-reversion times. B) Point estimates (connected dots) of cumulative incidences in Manaus. Included are the bootstrapped medians (crosses) and 95% confidence regions (shaded areas) using 1000 bootstrap samples.

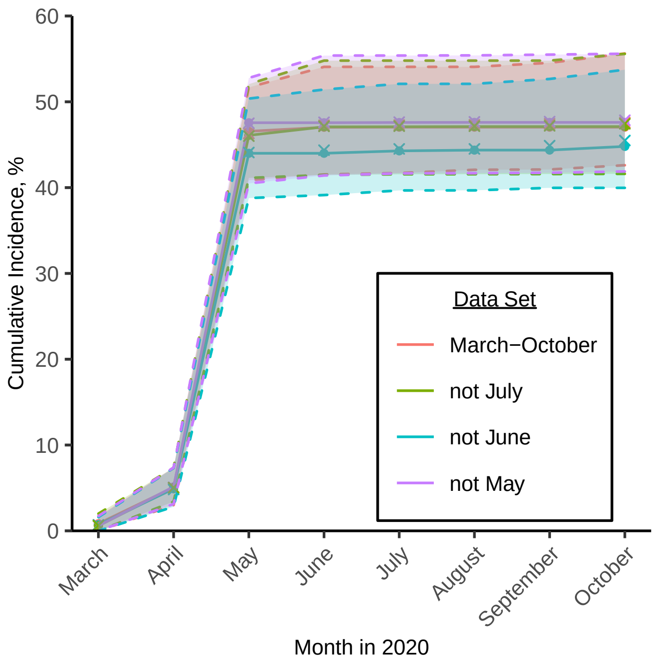

Web Figure 17: Point estimates (connected dots) of cumulative incidences in Manaus when using data from all serosurveys (March through October) or when dropping data from a single serosurvey. Also included are the bootstrapped medians (crosses) and 95% confidence regions (shaded areas) using 1000 bootstrap samples.

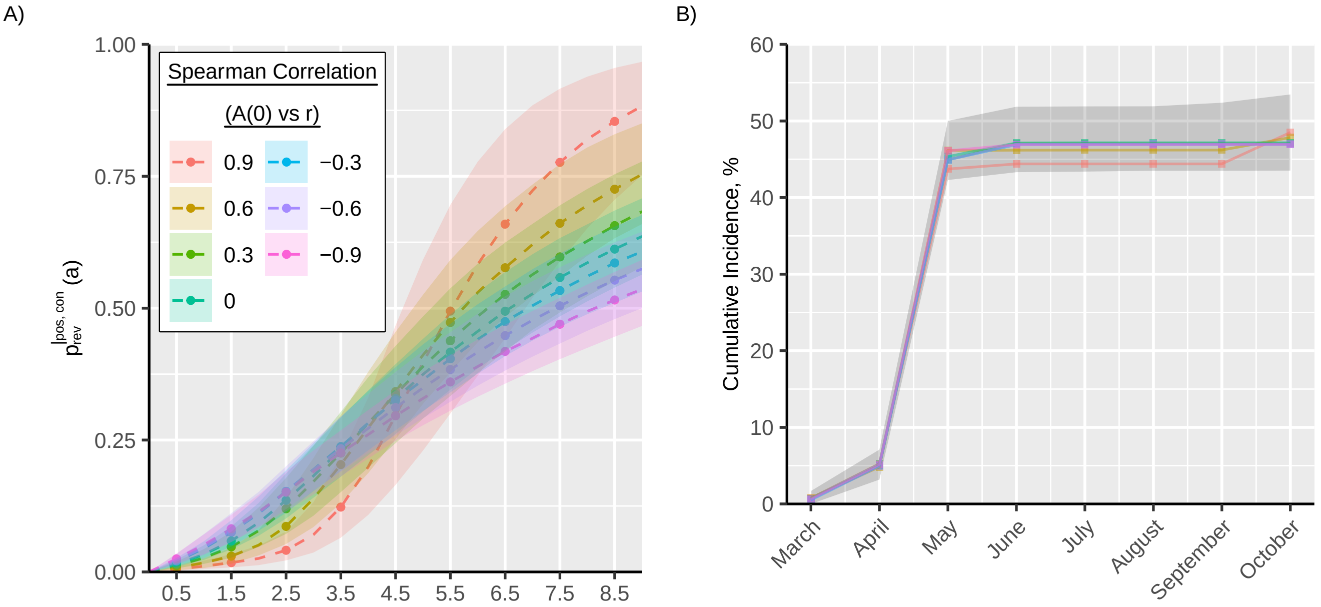

Web Figure 18: A) Probabilities to sero-revert within a given time past recovery when enforcing varying correlations between antibody peaks and decay rates. Shaded regions are bounded by the 2.5 and 97.5% quantiles obtained when resampling and rearranging the validation data to mimic the given correlation 100 times. Lines and dots show the median of the probabilities obtained from the 100 iterations. B) Cumulative incidence estimates using the empirically derived sero-reversion times when assuming various levels of correlation between anti-N IgG peak and decay rate. Also included is the 95% confidence region when no specific correlation is enforced (shaded gray region).
